# RITA modulates cell migration and invasion by affecting focal adhesion dynamics

**DOI:** 10.1002/1878-0261.12551

**Published:** 2019-08-06

**Authors:** Samira Catharina Hoock, Andreas Ritter, Kerstin Steinhäuser, Susanne Roth, Christian Behrends, Franz Oswald, Christine Solbach, Frank Louwen, Nina‐Naomi Kreis, Juping Yuan

**Affiliations:** ^1^ Department of Gynecology and Obstetrics, School of Medicine J. W. Goethe‐University Frankfurt Germany; ^2^ Institute of Biochemistry II, Medical School J. W.‐Goethe University Frankfurt Germany; ^3^ Department of Internal Medicine I, Center for Internal Medicine Medical Center Ulm Germany; ^4^ Present address: Solvadis Distribution GmbH Gernsheim Germany; ^5^ Present address: Munich Cluster of Systems Neurology Ludwig‐Maximilians‐Universität München Munich Germany

**Keywords:** FAK, focal adhesion, integrin, invasion, LPP, RITA

## Abstract

RITA, the RBP‐J interacting and tubulin‐associated protein, has been reported to be related to tumor development, but the underlying mechanisms are not understood. Since RITA interacts with tubulin and coats microtubules of the cytoskeleton, we hypothesized that it is involved in cell motility. We show here that depletion of RITA reduces cell migration and invasion of diverse cancer cell lines and mouse embryonic fibroblasts. Cells depleted of RITA display stable focal adhesions (FA) with elevated active integrin, phosphorylated focal adhesion kinase, and paxillin. This is accompanied by enlarged size and disturbed turnover of FA. These cells also demonstrate increased polymerized tubulin. Interestingly, RITA is precipitated with the lipoma‐preferred partner (LPP), which is critical in actin cytoskeleton remodeling and cell migration. Suppression of RITA results in reduced LPP and α‐actinin at FA leading to compromised focal adhesion turnover and actin dynamics. This study identifies RITA as a novel crucial player in cell migration and invasion by affecting the turnover of FA through its interference with the dynamics of actin filaments and microtubules. Its deregulation may contribute to malignant progression.

**Abbreviations**
FA: focal adhesion
F‐actin: filamentous actin
LPP: lipoma‐preferred partner
MEFs: mouse embryonic fibroblasts
MT: microtubule
RITA: RBP‐J‐interacting and tubulin‐associated protein

## 1 Introduction

Aberrant cell migration contributes to cancer metastasis responsible for over 90% of cancer associated deaths (Chaffer and Weinberg, 2011). Cell migration is precisely regulated by a controlled turnover of cellular anchors characterized by four distinct events: leading edge protrusion with dynamic actin polymerization, adhesion to the extracellular matrix (ECM), generation of contraction stress against adhesions, and the release of adhesions through disassembly and actin depolymerization (Gardel *et al.*, 2010; Parsons *et al.*, 2010). Adhesions are mediated by a large family of cell surface receptors formed by integrin dimers (Guo and Giancotti, 2004). The most common forms are focal adhesions (FA), composed of 232 different proteins forming the integrin adhesome network with over 6500 interactions demonstrating its complexity (Winograd‐Katz *et al.*, 2014). Integrins transduce signals through co‐clustering and recruitment of numerous scaffolding proteins including the cytoplasmic nonreceptor tyrosine kinase focal adhesion kinase (FAK), which regulates intracellular pathways like migration (Guo and Giancotti, 2004). Besides the orchestrated formation of FAs, spatial and temporal control of their assembly and disassembly is necessary for migration to allow cells to move in a directed fashion (Webb *et al.*, 2002). FA turnover also requires dynamic microtubules (MTs), their associated proteins, and coordinated interaction with the actin cytoskeleton (Etienne‐Manneville, 2013; Stehbens and Wittmann, 2012). Since the underlying mechanisms are not completely understood, elucidation of the regulation of FA dynamics by MT‐associated proteins (MAPs) will extend and refine the understanding of cancer cell migration and metastasis.

RITA, the RBP‐J‐interacting and tubulin‐associated protein, is a newly identified MAP and modulator of MT dynamics by coating MTs and affecting their stability in addition to its role in the Notch signaling pathway (Steinhauser *et al.*, 2017; Wacker *et al.*, 2011). Our previous work showed that suppression of RITA leads to more stable mitotic MTs associated with increasing numbers of chromosomal errors in mitosis (Steinhauser *et al.*, 2017). Interestingly, elevated RITA expression is correlated with unfavorable clinical outcome in anal carcinoma treated with concomitant chemoradiotherapy (Rodel *et al.*, 2018). However, both overexpression and downregulation of RITA have been reported in primary malignant tumor entities (Rodel *et al.*, 2018; Wang *et al.*, 2013), suggesting that RITA must be precisely regulated and its deregulation could influence malignant progression. It remains to be delineated which cellular events are affected by RITA. In the present work, we investigate the function of RITA in cell migration with an emphasis on adhesion dynamics. We show that knockdown of RITA impairs FA dynamics leading to decreased migration and invasion of diverse cancer cell lines as well as mouse embryonic fibroblasts (MEF).

## 2 Materials and methods

### 2.1 MEFs, cell culture, stable cell lines, transfection, and DNA constructs

The generation of RITA knockout mice, MEF isolation, and genotyping was previously described (Kreis *et al.*, 2019b; Steinhauser *et al.*, 2017). The phenotype analysis of knockout mice is under investigation. All experiments were performed in compliance with the German animal protection law, with institutional guidelines and approved by the ‘Tierforschungszentrum’ (TFZ), University of Ulm.

MDA‐MB‐231, MCF‐7, HeLa, and HEK293 cells were cultured as instructed by the supplier (ATCC, Wesel, Germany). HeLa cells stably expressing shGFP or shRITA were cultured in selective medium containing G418 (1.5 mg·mL^−1^) obtained from Invitrogen (Karlsruhe, Germany). The shRNA fragment targeting human RITA was generated by annealing sense oligonucleotide 5′‐GATCC‐GCTG CCA AGT GCG AAT AAA CGT TCA TAT GGC GTT TAT TCG CAC TTG GCA GCT TTT TA‐3′ and its antisense oligonucleotide (shRNA RITA). The control shRNA cell line shGFP was generated as described (Kreis *et al.*, 2009). After digestion with *Bam*HI and *Hin*dIII, the fragments were inserted into a phH1 vector, derived from a pEGFP‐C2 vector (BD Biosciences, Heidelberg, Germany) (Kreis *et al.*, 2009). Transfection, selection, and analysis of clones were performed as reported (Kreis *et al.*, 2009). Colony PCR analysis was performed using the sense primer 5′‐GCA GAT CGG ATC CAG TAA GAC CCC CGT GGA GCT G‐3′ and its antisense primer 5′‐GTC TTT GGT TTC GGG GGA ACC TTT ACT CTT AGG TTA A‐3′. Genomic DNA from nontransfected HeLa cells or plasmid DNA (phH1/shRNA RITA) was used as negative and positive control. siRNA against the coding region or the 3′ UTR of RITA is GGAAGAAGAACAAAUACAG (siRITA) or AGGGAACCCCAGGUAUUAAUU (siRITA‐UTR), respectively, obtained from Sigma‐Aldrich, Taufkirchen, Germany. Control siRNA was from Qiagen (Hilden, Germany). siRNA (10–20 nm) were transiently transfected with Oligofectamine^TM^ (Thermo Fisher Scientific, Dreieich, Germany), as reported (Kreis *et al.*, 2016). Cloning of GFP‐full length RITA was previously described (Wacker *et al.*, 2011). pCMV‐HA vector was from Clontech Laboratories (Mountain View, CA, USA) using *Eco*RI und *Xho*I cloning sites. DNA was transfected as reported (Kreis *et al.*, 2014).

### 2.2 Cell viability, wound healing/migration, invasion assay, and gene analysis

Cell proliferation assays were performed using Cell Titer‐Blue® Cell Viability Assay (Promega, Mannheim, Germany) as described (Kreis *et al.*, 2015). Cell migration/wound healing assays were performed with culture inserts from ibidi (Ibidi GmbH, Martinsried, Germany). Culture inserts (cell‐free gap of 500 μm) were placed in a 6‐cm culture dish, and both wells were filled with cell suspensions (MCF‐7, 6.5 × 10^4^; MDA‐MB‐231, 7.5 × 10^4^). After 14 h, the culture inserts (in triplicate for each condition) were removed and the images were obtained at indicated time points. Cell‐free area was evaluated based on bright‐field images using the axiovision SE64 Re. 4.9 software (Zeiss, Jena, Germany); for each experiment, at least 12 migration front images were taken and analyzed, and the experiments were independently performed three times.

For invasion assay, cells were seeded in 24‐well transwell matrigel chambers according to the manufacturer’s instructions (Cell Biolabs Inc, San Diego, CA, USA), as reported (Ritter *et al.*, 2015). Briefly, cells (MDA‐MB‐231, 7.5 × 10^4^; MCF‐7, 12 × 10^4^; MEFs, 5 × 10^4^) were seeded into the upper chamber of the transwell in 500 μL serum‐free medium and the lower chamber was filled with 750 μL serum‐free medium. After 12 h, the medium of both chambers was discarded and the invasion assays were started by adding medium containing 10% FBS for the next 24 h. Cells were fixed with ethanol and stained with 4′,6‐diamidino‐2‐phenylindole‐dihydrochloride (DAPI). Invaded cells were counted with a microscope. The experiments were independently performed three times.

RNA extraction and real‐time PCR were performed as described (Kreis *et al.*, 2019a; Ritter *et al.*, 2018).

### 2.3 Cell motility evaluation via time‐lapse microscopy

Cells were seeded into 24‐well plates with a low confluence and imaged at 5‐min intervals for 12 h. Time‐lapse imaging was performed with an AxioObserver.Z1 microscope (Zeiss), imaged with an AxioCam MRc camera (Zeiss), and equipped with an environmental chamber to maintain proper environmental conditions (37 °C, 5% CO_2_). The movies were analyzed using imagej 1.49i software (National Institutes of Health, Bethesda, MD, USA) with the manual tracking plugin, and Chemotaxis and Migration Tool (Ibidi GmbH). Tracks were derived from raw data points and were plotted in graphpad prism 7 (GraphPad software Inc., San Diego, CA, USA). The accumulated distance was calculated using the raw data points by the Chemotaxis and Migration Tool. Thirty random cells per experiment were analyzed, and the experiments were repeated independently three times. The patterns of motility were evaluated, and migration velocity and directionality were calculated as described (Ritter *et al.*, 2016).

### 2.4 Western blot analysis, immunoprecipitation, and measurement of polymerized α‐tubulin

Western blot analysis and immunoprecipitation (IP) were performed as described (Kreis *et al.*, 2009; Muschol‐Steinmetz *et al.*, 2016). Cellular lysates were prepared using RIPA buffer [50 mm Tris pH 8.0, 150 mm NaCl, 1% NP‐40, 0.5% Na‐deoxycholate, 0.1% SDS, 1 mm NaF, 1 mm DTT, phosphatase, and protease inhibitor cocktail tablets (Roche, Mannheim, Germany)]. About 600–800 µg of cellular lysates were used for IP. For Flag‐IP, Flag®‐M2 affinity gel (Sigma‐Aldrich) was added to the lysates and incubated on a rotator at 4 °C overnight. The precipitates were washed three times before SDS/PAGE. Following antibodies were used for western blot analysis, and IP: mouse monoclonal antibodies against α‐tubulin and Flag were from Sigma‐Aldrich; rabbit monoclonal antibody against pFAK (Tyr397) and polyclonal antibody against FAK were from Cell Signaling (Frankfurt, Germany). Rabbit polyclonal antibody against lipoma‐preferred partner (LPP) was from Enzo Life Science, Lausen, Switzerland. The RITA antibody was designed and commercially produced (rabbit monoclonal IgG; Epitomics, Burlingame, CA, USA), as described (Steinhauser *et al.*, 2017).

For the measurement of polymerized α‐tubulin *in vivo*, cells were transiently or stably depleted of endogenous RITA and stained for α‐tubulin for flow cytometry (Becton Dickinson, Heidelberg, Germany). Briefly, cellular soluble tubulin was pre‐extracted with a MT‐stabilizing buffer (2 mm EGTA, 5 mm MgCl_2_, 0.1 m PIPES pH 7.4, and 25 nm paclitaxel). Resuspended cells were then fixed with an equal volume of 4% paraformaldehyde solution at 37 °C for 15 min. Cells were washed, stained for α‐tubulin with a specific mouse monoclonal antibody (Sigma‐Aldrich) and FITC‐conjugated rabbit anti‐mouse antibody (Dako, Hamburg, Germany), and analyzed using a FACSCalibur™ (BD Biosciences). More than 95% of cells were included in the acquisition gate, and 30 000–50 000 cells were examined. Fluorescence intensity was quantified using the cell quest software (Becton Dickinson). The polymer content in control siRNA transfected cells was assigned as 100%.

### 2.5 Immunofluorescence staining and measurement

For indirect immunofluorescence staining, cells were fixed with 4% paraformaldehyde containing 0.1–0.2% Triton X‐100 for 15 min at room temperature (Kreis *et al.*, 2016). The following primary antibodies were used: rabbit polyclonal antibody against LPP (Enzo Life Science), mouse monoclonal antibody against pFAK (Cell Signaling), mouse monoclonal antibodies against paxillin (BD Transduction Laboratories^TM^) and α‐actinin (sarcomeric; Sigma‐Aldrich), and rat monoclonal antibodies against active β1‐integrin (CD29, clone 9EG7) and inactive β1‐integrin (CD29, clone Mab13; BD Pharmingen™, San Jose, CA, USA). FITC‐, Cy3‐, and Cy5‐conjugated secondary antibodies were obtained from Jackson Immunoresearch (Cambridgeshire, UK). DNA was stained using DAPI (Roche). The filamentous actin (F‐actin) cytoskeleton was stained using phalloidin (Phalloidin‐Atto 550; Sigma‐Aldrich). Slides were examined with an AxioObserver.Z1 microscope (Zeiss, Göttingen, Germany), and images were taken via an AxioCam MRm camera (Zeiss). The slides were further examined by confocal laser scanning microscopy using Z‐stack images with a HCXPI APO CS 63.0 × 1.4 oil objective (Leica CTR 6500, Heidelberg, Germany) in sequential excitation of fluorophores. A series of Z‐stack images were captured at 0.5‐μm intervals. All images in each experiment were taken with the same wave intensity and exposure time. All experiments, unless otherwise indicated, were independently performed at least three times. Representatives shown in figures are generated by superimposing (overlay) individual images from confocal Z‐sections. Mean fluorescence intensities for pFAK and F‐actin (phalloidin) were analyzed with LAS AF Lite (Leica); mean signal sizes, mean gray intensities of paxillin, inactive/active integrin, LPP and α‐actinin, and positive cell area were analyzed with NIH ImageJ. Co‐localization analyses were performed using NIH imagej Plugin JACop.

### 2.6 Nocodazole washout experiments, cell spreading/adhesion assay, and integrin measurement

To analyze the dynamics of FAs (FA disassembly/nocodazole washout assay), cells were treated with nocodazole (10 µm; Sigma‐Aldrich) for 5 h to depolymerize MTs (Ezratty *et al.*, 2005). The drug was washed out with PBS, and MTs were repolymerized in medium for different time points. For cell spreading/adhesion assays, four‐chamber slides were coated with 2 µg·mL^−1^ fibronectin for 15 min (Merck Millipore, Darmstadt, Germany) as described (Lilja *et al.*, 2017). Cells were trypsinized, reseeded on fibronectin‐coated slides (HeLa shGFP and shRITA, 3.5 × 10^4^ and MDA‐MB‐231, 4.5 × 10^4^ cells per chamber), and incubated at 37 °C for indicated time points. Unattached cells were removed by washing with PBS. Adherent cells were fixed with paraformaldehyde containing Triton X‐100 and stained. Quantification of cell area from slides released at 20 and 60 min postplating was analyzed using imagej. Antibody‐based analysis of active cell surface β1‐integrin was measured through flow cytometry (FACSCalibur™; BD Biosciences). To evaluate the dynamics of the actin cytoskeleton in the absence of RITA, cells were starved in serum‐free medium for 10 h. Cells were treated with latrunculin B (200 nm; BIOMOL GmbH, Hamburg, Germany) for 90 min to depolymerize actin fibers. The drug was washed out with PBS, and the cells were released into serum‐containing medium for indicated time points. The cells were fixed with 4% paraformaldehyde containing 0.2% Triton X‐100 for 15 min at room temperature before processing for immunofluorescence staining.

### 2.7 Mass spectrometry

Anti‐HA IP and HA peptide elution were performed followed by liquid chromatography coupled with tandem mass spectrometry (LC‐MS/MS) on trypsinized immune complexes (Behrends *et al.*, 2010). Peptide samples were separated on a nanoflow HPLC system (Thermo Scientific) and analyzed on a LTQ Orbitrap Elite (Thermo Scientific). Spectra were identified by Sequest searches followed by target‐decoy filtering and linear discriminant analysis as described (Behrends *et al.*, 2010). Comparative Proteomics Analysis Software Suite (CompPASS, http://pathology.hms.harvard.edu/labs/harper/Welcome.html) analysis was performed, and normalized and weighted D scores (WD^N^ scores) were calculated based on average peptide spectral matches (APSMs). Proteins with APSM ≥ 2 and WD^N^ ≥ 1 were generally considered as high‐confidence interaction partners (HCIPs) (Behrends *et al.*, 2010; Sowa *et al.*, 2009). To focus on the most hot spots, we have defined APSM ≥ 5 and WD^N^ ≥ 3 as HCIPs in this work.

### 2.8 Statistical analysis

Student’s *t*‐test (two tailed and paired or homoscedastic) was used to evaluate the significant difference between diverse groups for cell viability, invasion assay, fluorescence quantification of in/active integrin and pFAK, spreading assay, Pearson correlation, and FACS measurements of polymerized α‐tubulin and active integrin. Statistical evaluation of single cell tracking, line‐scan analysis [LPP, α‐actinin, paxillin, active integrin (without spreading)], latrunculin B washout, and the measurement of cell area was performed by using an unpaired Mann–Whitney *U*‐test (two tailed). Difference was considered as statistically significant when *P* < 0.05. Manders’ M1 & M2 coefficients vary between 0 and 1, from nonoverlapping images to 100% co‐localization between two images (Manders *et al.*, 1993). M1 is defined as the ratio of the ‘summed intensities of pixels from the green image for which the intensity in the red channel is above zero’ to the ‘total intensity in the green channel’, and M2 is defined conversely for red (Manders *et al.*, 1993; Zinchuk *et al.*, 2007). The Pearson’s coefficient is interpreted as following: A value of +1 is defined as a positive linear relationship, whereas a value of −1 is considered as a negative one. The value 0 is regarded as no linear relationship.

## 3 Results

### 3.1 Depletion of RITA reduces cell motility of cancer cells and MEFs

**Figure 1:**
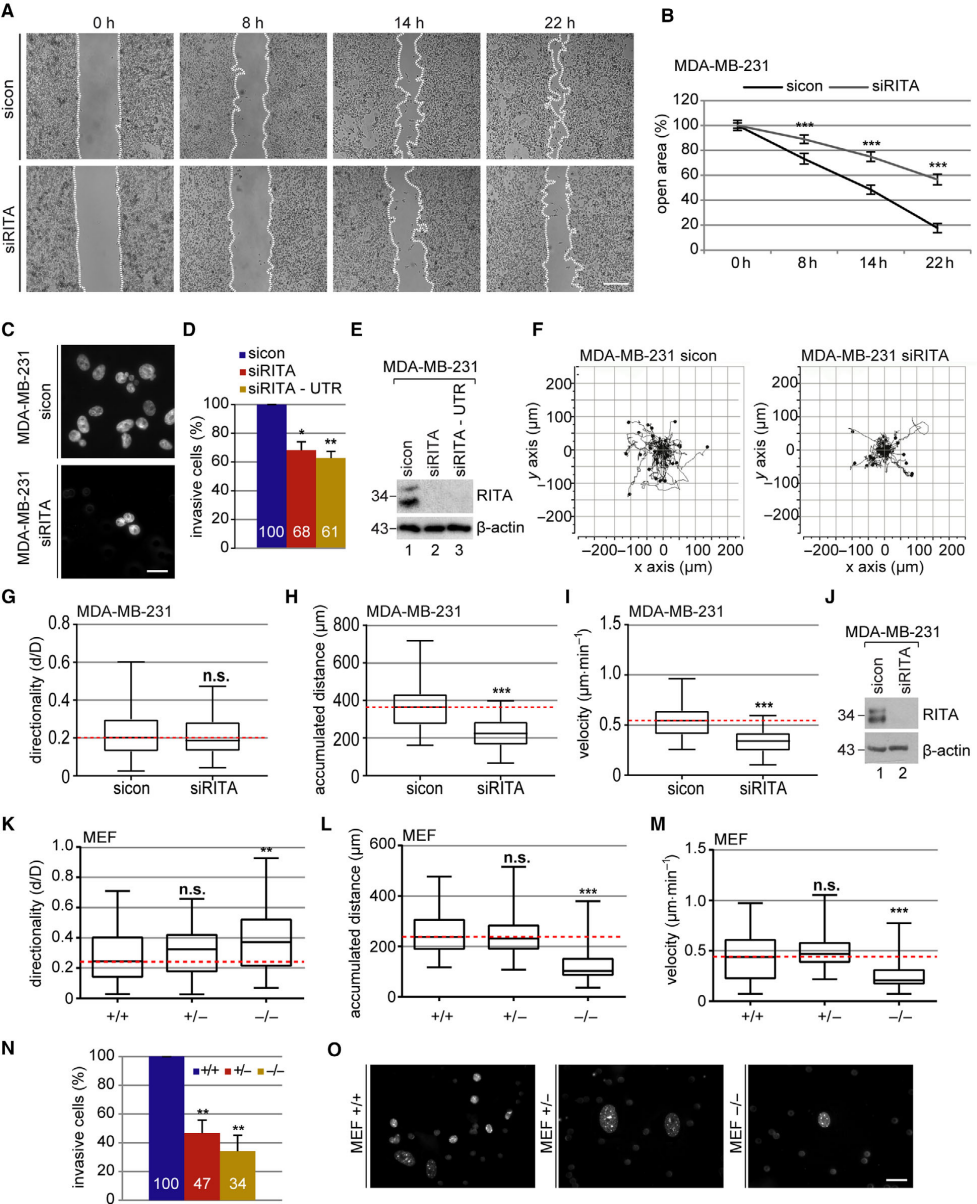
Decreased migration and invasion of breast cancer cell lines and MEFs lacking RITA. (A) Wound healing/migration assays were performed with MDA‐MB‐231 cells, and images were taken at indicated time points to document the migration front. Representatives are shown. White dashed line depicts the migration front. Scale: 300 μm. (B) Quantification of the open area between both migration fronts at various time points. The cell‐free area at 0 h was assigned as 100%. The results from three independent experiments are presented as mean ± SEM. ***P < 0.001. (C) Invasion assay. MDA‐MB‐231 cells were treated with control siRNA (sicon), siRNA against the coding region (siRITA) or the 3′ UTR of RITA (siRITA‐UTR); seeded into transwell systems; and starved for 12 h. The cells were released into fresh medium for 24 h and fixed for quantification. Representatives of invaded MDA‐MB‐231 cells are shown. Scale: 25 μm. (D) Quantification of invaded cells. The total number of invaded cells in the control group was assigned as 100%. The results from three independent experiments are presented as mean ± SEM. *P < 0.05, **P < 0.01. (E) Control western blot analysis showing the efficient knockdown of endogenous RITA in MDA‐MB‐231 cells treated with sicon, siRITA, or siRITA‐UTR. β‐actin served as a loading control. (F–I) Time‐lapse microscopy was performed with sicon or siRITA‐treated MDA‐MB‐231 cells for up to 12 h. Random motility of these cells was analyzed. Representative trajectories of individual cells (*n* = 30) are shown (F). Evaluated directionality (G), accumulated distance (H), and velocity (I) from three independent experiments are shown as box plots with variations. ***P < 0.001. (J) Control western blot analysis shows the efficient knockdown of endogenous RITA in MDA‐MB‐231 cells. β‐actin served as a loading control. (K–M) MEFs were subjected to time‐lapse microscopy to analyze their random motility. Evaluation of directionality (K), accumulated distance (L), and velocity (M) from three individual experiments is depicted. **P < 0.01, ***P < 0.001. (N) Invasion assay. The total number of invasive wild‐type MEFs was assigned as 100%. The results from three independent experiments are presented as mean ± SEM. **P < 0.01, compared to wild‐type MEFs. (O) Representatives of invaded MEFs are shown. Scale: 25 μm. Student’s *t*‐test for (B), (D), and (N). Unpaired Mann–Whitney *U*‐test for (G–I) and (K–M).

To determine whether RITA influences the migration capacity of cancer cells, wound healing/migration assays were performed with the highly metastatic breast cancer cell line MDA‐MB‐231 (Fig. S1A). MDA‐MB‐231 cells treated with siRNA targeting the coding region of RITA (siRITA; Fig. 1E) or its 3′ UTR (siRITA‐UTR; Figs 1E and S3H) showed a significant decreased migration rate compared to control cells (Fig. 1A,B; Fig. S1B). These results were corroborated with the low malignant breast cancer cell line MCF‐7 (Fig. S1C–E). Although depletion of RITA resulted in impaired migration, overexpression of Flag‐RITA had hardly impact on the migration capacity of MCF‐7 cells (Fig. S1H–J).

To underline the observations, invasion assays were performed in both breast cancer cell lines. Relative to control siRNA‐treated MDA‐MB‐231 cells, knockdown of RITA with siRITA or siRITA‐UTR reduced the number of cells passing the matrigel by 32% or 39% (Fig. 1C,D). MCF‐7 cells depleted of RITA showed a reduced number of passing cells even by 66% compared to control cells (Fig. S1F,G). Moreover, single MDA‐MB‐231 cells were tracked by time‐lapse microscopy and their migratory parameters were evaluated (Fig. 1F–J). Whereas the directionality was scarcely changed (Fig. 1G), their accumulated distance and velocity were significantly decreased in siRITA‐treated cells compared to control cells (Fig 1H,I). Comparable results were obtained with MDA‐MB‐231 cells depleted of RITA with siRITA‐UTR (Fig S2A–C, Fig. S3H).

To exclude the possibility that knockdown of RITA alters cell viability affecting thus migration behavior, cell titer blue assay was performed. There was barely any difference in the proliferation rate between MDA‐MB‐231 and MCF‐7 cells depleted of RITA and their control cells (Fig. S2D–H). Additionally, we tested whether Flag‐RITA overexpression influences proliferation of MCF‐7 cells. Our results indicate no changes between cells overexpressing RITA and control cells (Fig. S2I,J).

To investigate this issue in depth, knockout mice were generated and MEFs were isolated from RITA wild‐type (+/+), heterozygous (+/−), and homozygous (−/−) embryos (Steinhauser *et al.*, 2017) for single cell tracking. Interestingly, relative to RITA wild‐type and heterozygous MEFs, RITA^−/−^ MEFs displayed a remarkably decrease in the accumulated distance and velocity, and an increase in the directionality (Fig. 1K–M). Furthermore, invasion assays were performed with MEFs. RITA^+/−^ and RITA^−/−^ MEFs showed a significant decrease in the number of invaded cells compared to RITA wild‐type MEFs (Fig. 1N,O). These data provide strong evidence that RITA is involved in the modulation of cell motility of malignant and primary cells.

### 3.2 Increased FA proteins and FA size in RITA‐depleted cells

Integrins are crucial for cell migration by coupling the ECM to the actin cytoskeleton within FAs (Geiger *et al.*, 2001). They exist in a low‐affinity inactive state and a high‐affinity active state, which can be detected using a specific antibody recognizing the active β1‐integrin conformation (9EG7; Lilja *et al.*, 2017). To determine whether RITA is involved in the formation of FAs, HeLa cell lines stably expressing shRNA against GFP or RITA were generated (HeLa shGFP and HeLa shRITA). RITA was efficiently depleted in HeLa shRITA cells (Fig. S3A). In comparison with HeLa shGFP cells, these cells demonstrated reduced migration (Fig. S3B), invasion (Fig. S3C), and motility (Fig. S3D–F).

**Figure 2:**
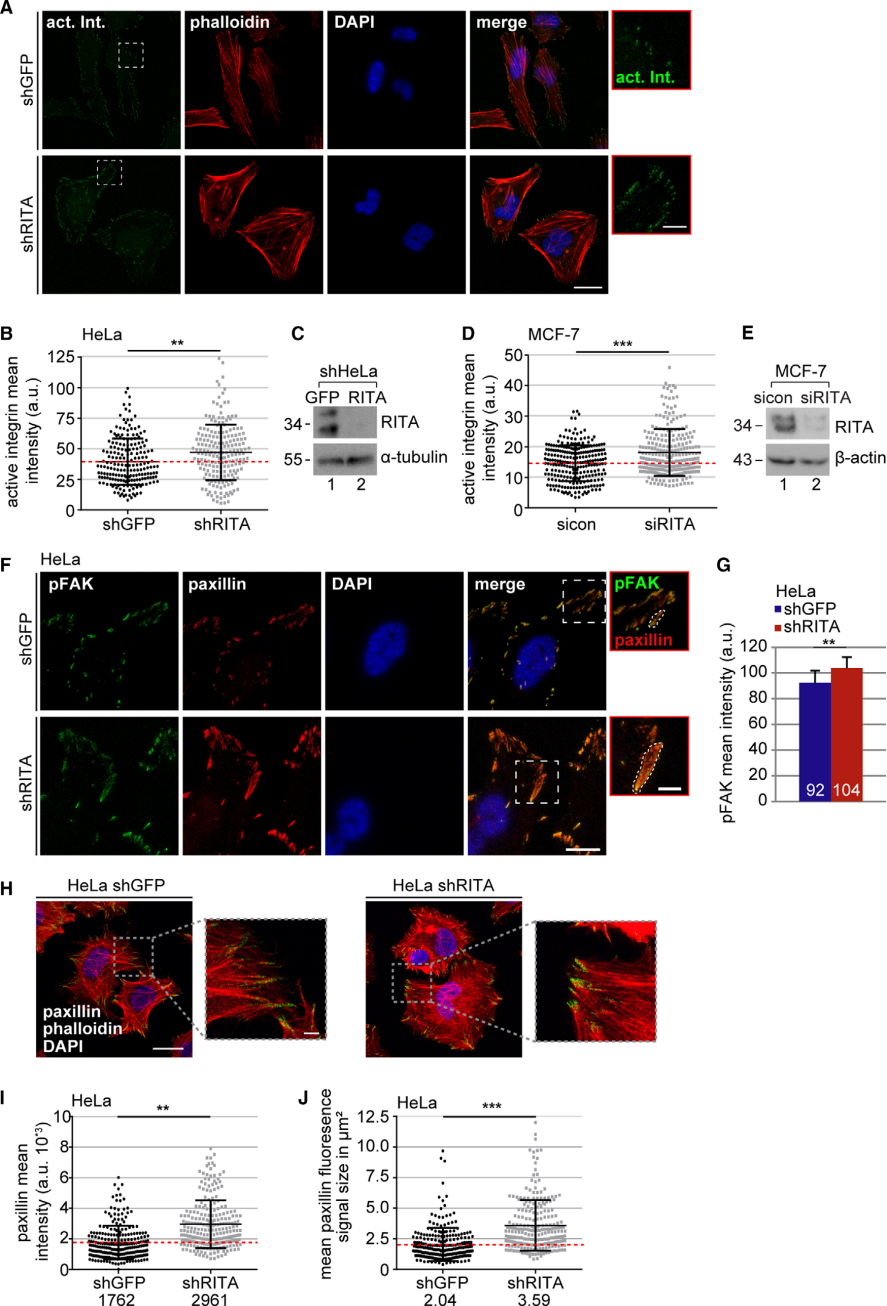
Suppression of RITA enhances various FA proteins. (A) HeLa cells stably expressing shGFP or shRITA were stained as indicated for fluorescence microscopy. Representative images are shown. Scale: 25 µm; inset scale: 5 µm. (B) Quantification of the mean fluorescence intensity of active β1‐integrin (9EG7; green in A) at FAs (240 FAs) in a defined region of interest (ROI). The results from three independent experiments are presented as scatter plots with bars indicating varied intensities. **P < 0.01. a.u., arbitrary units. (C) Control western blot analysis showing the efficient knockdown of RITA. α‐tubulin served as a loading control. (D) As described in (A), MCF‐7 cells were transfected with sicon or siRITA and stained for fluorescence microscopy (images not shown). The mean fluorescence intensity of active β1‐integrin (9EG7) at FAs (240 FAs) in a defined ROI was measured. The results from three independent experiments are presented as scatter plots with bars indicating varied intensity ranges. ***P < 0.001. a.u., arbitrary units. (E) Control western blot analysis showing the efficient knockdown of RITA. β‐actin served as loading control. (F) shHeLa cells were stained for pFAK (green), paxillin (red), and DNA (DAPI, blue). Representatives are shown. Scale: 25 µm; inset scale: 5 µm. (G) Quantification of the mean fluorescence intensity of pFAK in shHeLa cells in a defined ROI (240 FAs). The results are based on three independent experiments and presented as mean ± SEM. **P < 0.01. (H) HeLa shGFP and shRITA cells were stained for paxillin (green), F‐actin (phalloidin, red), and DNA (DAPI, blue) for fluorescence microscopy. Representatives are shown. Regions outlined in boxes are shown in a higher magnification. Scale: 25 µm; insets: 5 µm. (I) Quantification of the mean gray intensity of the outlined paxillin signal in shHeLa cells (240 FAs). The results from three independent experiments are presented as scatter plots with bars indicating varied intensity ranges. **P < 0.01. a.u., arbitrary units. (J) Measurement of the FA size of shHeLa cells based on the outlined fluorescence signal of paxillin from (F) as depicted in insets. The results from three independent experiments (240 FAs) are presented as scatter plots with bars indicating varied sizes in µm^2^. Unpaired Mann–Whitney *U*‐test for (B), (D), and (I–J). Student’s *t*‐test for (G).

To look at the activation of integrin, HeLa shGFP and HeLa shRITA cells (Fig. 2C) were stained for active integrin, F‐actin (phalloidin), and DNA for microscopy. Unexpected, relative to HeLa shGFP cells, the active integrin was more demonstrative in HeLa shRITA cells (Fig. 2A). The mean intensity of active integrin was further measured on the cell surface. This analysis revealed more active integrin in cells stably knocked down of RITA (Fig. 2B). As RITA is a negative modulator of the Notch pathway, we asked whether its knockdown could increase the gene expression of integrin. Interestingly, compared to HeLa shGFP cells, the mRNA level of integrin was even lowered, though not significantly, in HeLa shRITA cells (Fig. S3G), indicating that this increased signal of active integrin is not attributable to its gene expression. The mean intensity of active integrin was also increased in MCF‐7 cells transiently depleted of RITA with siRNA (Fig 2D,E).

Integrin clustering causes FAK autophosphorylation affecting cell motility (Kornberg *et al.*, 1992). To study this issue, HeLa shGFP and HeLa shRITA cells were stained for FAK phosphorylated at Y397 (pFAK). The stable knockdown of RITA led to an increase of 12% in pFAK intensity (Fig. 2F,G). Additionally, we stained these cells for paxillin, a FA adaptor, and scaffolding protein, which is phosphorylated partially by FAK upon integrin clustering (Lopez‐Colome *et al.*, 2017). The mean intensity of paxillin was significantly increased by 68% at FAs of HeLa shRITA cells compared to HeLa shGFP cells (Fig. 2F,H,I). Moreover, recent studies suggest that the FA size uniquely predicts cell migration speed (Kim and Wirtz, 2013). To address this, the size of FAs was measured based on the paxillin staining. This measurement demonstrated that the FA size was enlarged up to 76% with an average size of 3.59 µm^2^ in HeLa shRITA cells in comparison with 2.04 µm^2^ in HeLa shGFP cells (Fig. 2J).

**Figure 3:**
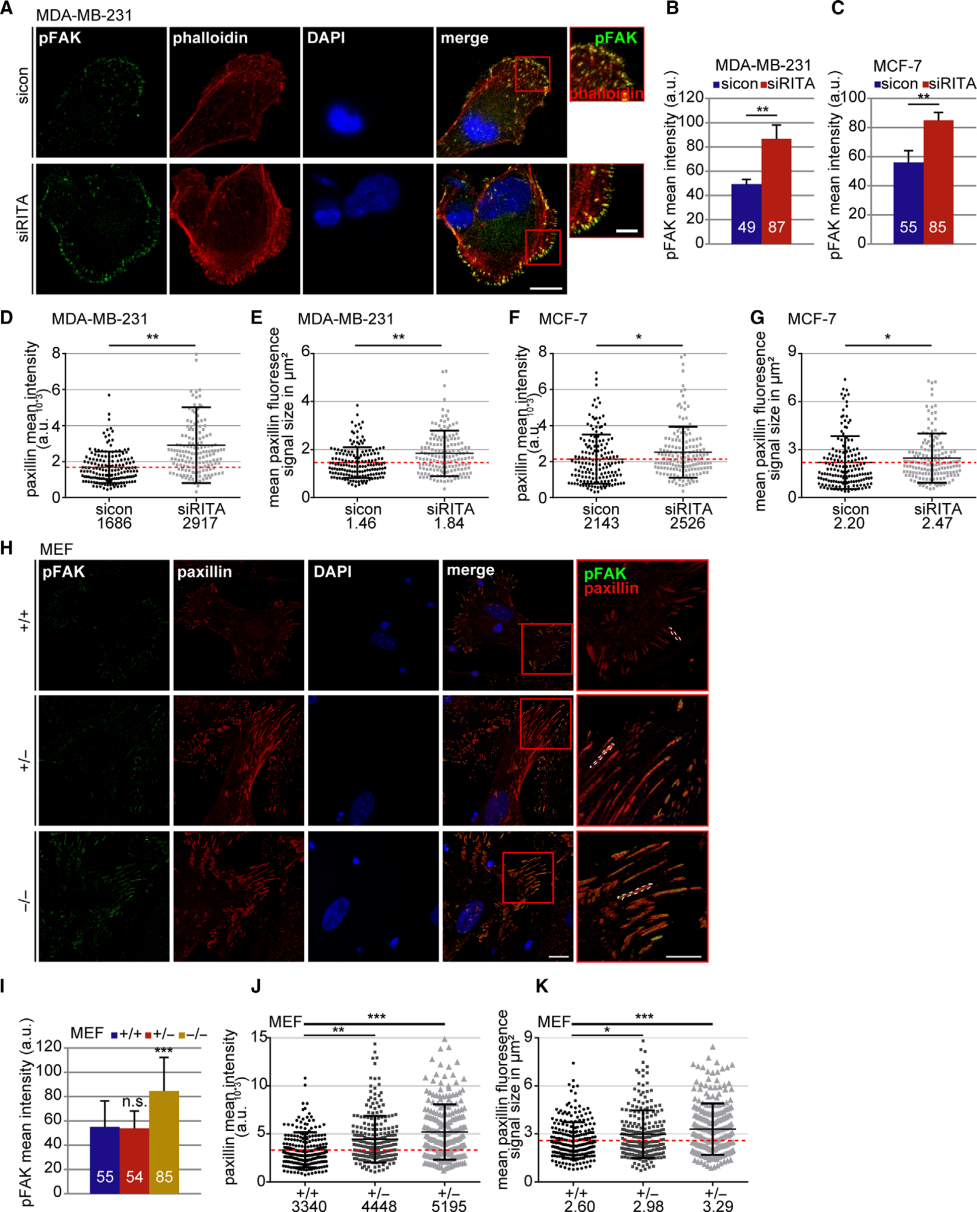
Depletion of RITA increases the amount of pFAK in cells. (A) MDA‐MB‐231 cells were treated with sicon and siRITA and stained for pFAK (green), F‐actin (phalloidin, red), and DNA (DAPI, blue) for fluorescence microscopy. Representative images are shown. Scale: 12.5µm; inset scale: 5 µm. (B) Quantification of the mean fluorescence intensity of pFAK in MDA‐MB‐231 cells in a defined ROI (210 FAs). The results are based on three independent experiments and presented as mean ± SEM. **P < 0.01. a.u., arbitrary units. (C) Quantification of the mean fluorescence intensity of pFAK in MCF‐7 cells in a defined ROI (210 FAs). The results are based on three independent experiments and presented as mean ± SEM. **P < 0.01. (D) Quantification of the mean gray intensity of the outlined paxillin signal in MBA‐MB‐231 cells (160 FAs). The results are based on two independent experiments and presented as scatter plots with bars indicating varied intensity ranges. **P < 0.01. a.u., arbitrary units. (E) Measurement of the FA size in MDA‐MB‐231 cells based on the outlined fluorescence signal of paxillin (images not shown). The results from two independent experiments (160 FAs) are presented as scatter plots with bars indicating varied sizes in µm^2^. **P < 0.01. (F) Quantification of the mean gray intensity of the outlined paxillin signal in MCF‐7 cells (160 FAs). The results are based on three independent experiments (240 FAs) and presented as scatter plots with bars indicating varied intensity ranges. *P < 0.05. a.u., arbitrary units. (G) Measurement of the FA size in MCF‐7 cells based on the outlined fluorescence signal of paxillin (images not shown). The results from three independent experiments (240 FAs) are presented as scatter plots with bars indicating varied sizes in µm^2^. *P < 0.05. (H) Wild‐type (RITA^+/+^), heterozygous (RITA^+/−^), and homozygous (RITA^−/−^) MEFs were stained for pFAK (green), paxillin (red), and DNA (DAPI, blue) for fluorescence microscopy. Representatives are shown. Scale: 25 µm; inset scale: 20 µm. (I) Quantification of the mean fluorescence intensity of pFAK. The results are based on the measurement of 70 FAs per condition and presented as mean ± SD. ***P < 0.001. (J) Quantification of the mean gray intensity of the outlined paxillin signal in MEFs. The results are based on three independent experiments (240 FAs per condition) and presented as scatter plots with bars indicating varied intensity ranges. **P < 0.01, ***P < 0.001. a.u., arbitrary units. (K) Measurement of the FA size in MEFs based on the outlined fluorescence signal of paxillin. The results from three independent experiments (240 FAs) are presented as scatter plots with bars indicating varied sizes in µm^2^. Student’s *t*‐test for (B, C) and (I). Unpaired Mann–Whitney *U*‐test for (D–G) and (J–K).

In line with these data, MDA‐MB‐231 and MCF‐7 cells with transient siRITA knockdown showed elevated levels of pFAK in immunofluorescence staining (Fig. 3A–C) and western blot analysis compared to control cells (Fig. S4A–C). Increases in the mean intensity of paxillin and FA size were also observed in these cell lines (Fig. 3D–G).

In further support of these observations, an elevation in pFAK and paxillin intensities was also detected in MEFs RITA^+/−^ and RITA^−/−^ cells (Fig. 3H–J). While the FA size was mildly enlarged in RITA^+/−^ MEFs, it extended greater in RITA^−/−^ MEFs compared to RITA wild‐type MEFs (Fig. 3K). These results reinforce the notion that insufficiency of RITA is associated with increased FA proteins and enlarged FAs.

### 3.3 MT‐induced FA turnover is impaired in cancer cells deficient of RITA

**Figure 4:**
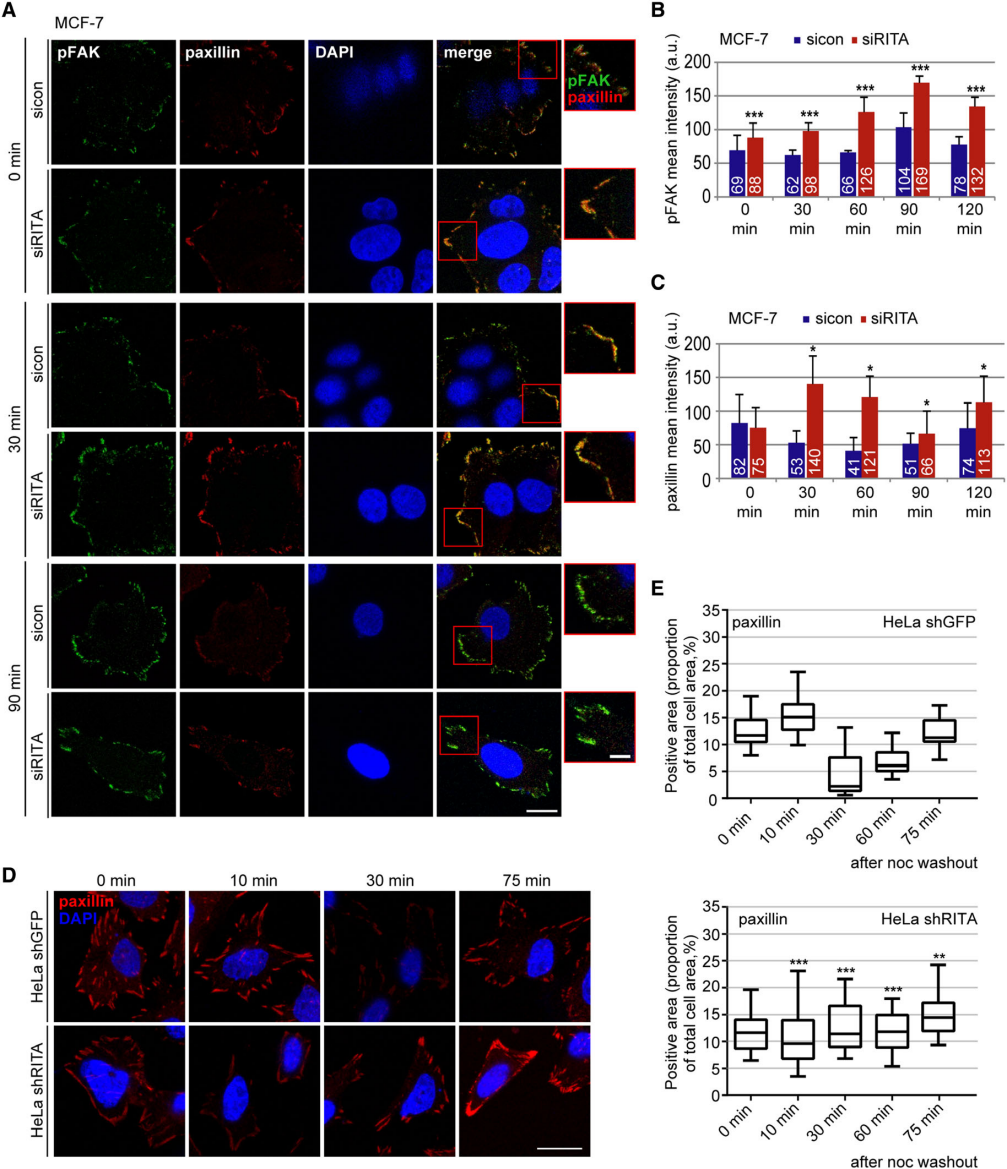
Deficiency of RITA delays the MT‐induced FA disassembly. (A) MCF‐7 cells were transfected with control siRNA or siRITA. The cells were incubated for 5 h with 10 µm nocodazole followed by washout, where the MTs were allowed to regrow for the indicated time points. Cells were stained for pFAK (green), paxillin (red), and DNA (DAPI, blue). Representatives of FA reassembly are shown. Scale: 12.5 µm, inset scale: 5 µm. (B, C) Kinetics of FA disassembly during MT regrowth after washout. Quantification of the mean fluorescence intensity of pFAK (B) and paxillin (C) (70 FA per condition in a defined ROI) is depicted. The results are based on three independent experiments and presented as mean ± SEM. *P < 0.05, ***P < 0.001. a.u., arbitrary units. (D) HeLa cells stably transfected with shGFP or shRITA were treated with 10 µm nocodazole for 5 h followed by washout for the indicated time points and stained for paxillin (red) and DNA (DAPI, blue). Representatives of FA reassembly are shown. Scale: 25 µm. (E) The fluorescence signals of the paxillin staining were measured and analyzed in relation to the total cell area (in percentage). The results are based on three independent experiments (240 FAs) and presented as box plots with bars indicating variations. Upper panel: HeLa shGFP cells, lower panel: HeLa shRITA cells. **P < 0.01, ***P < 0.001. Student’s *t*‐test for (B, C) and (E).

Coordinated assembly and disassembly of FAs is required for effective cell migration (Webb *et al.*, 2002). The decrease in migration speed and the increase in FA size argue for an impaired FA disassembly, which was analyzed in detail using a well‐established FA disassembly assay (Ezratty *et al.*, 2005). This assay is based on the treatment with nocodazole leading to MT depolymerization and stable FAs through stress fiber formation and followed by nocodazole washout triggering FA disassembly through rapid MT regrowth (Ezratty *et al.*, 2005). To study the potential effect of RITA on FA dynamics, MCF‐7 cells treated with control siRNA or siRNA targeting RITA (Fig. S4B) were subjected to nocodazole and released for 0, 30, 60, 90, and 120 min. Cells were stained for pFAK and paxillin, two important FA assembly factors, for microscopic evaluation (Fig. 4A). After nocodazole washout, while the pFAK signal kept relatively constant till 60 min, it was elevated at 90 min and marginally decreased at 120 min in control cells (Fig. 4B). By contrast, depletion of RITA enhanced the pFAK signal already at 0 min, which reached a peak at 90 min and slightly decreased at 120 min (Fig. 4B). In the case of paxillin, the signal was decreased at 30 and 60 min and raised at 90 and 120 min in control cells, whereas it peaked at 30 min, turned down at 60/90 min, and raised again at 120 min after the washout in RITA‐depleted cells (Fig 4A,C). These results suggest that reduction of RITA leads to a MT‐related disorganized disassembly and reassembly of FAs.

To underscore the results, HeLa shGFP and HeLa shRITA cells were treated with nocodazole; released for 0, 10, 30, 60, and 75 min; and stained for paxillin. Microscopic analysis revealed again that depletion of RITA impaired FA dynamics showing a relatively constant intensity of the paxillin signal throughout the kinetics (Fig. 4D,E, lower panel). Conversely, the paxillin signal in control cells ran a dynamic course evidenced by going down from 10 min, reaching the lowest point at 30 min and starting to accumulate the signal afterward (Fig. 4D,E, upper panel). Comparable results were also obtained from MDA‐MB‐231 cells depleted of RITA and stained for pFAK and paxillin (Fig. S4C–F). Though the disassembly and reassembly courses varied among cell lines, depending on individual cellular context, these results clearly indicate that suppression of RITA impairs the MT‐associated FA turnover.

### 3.4 RITA is involved in the regulation of MT and actin dynamics during the interphase

**Figure 5:**
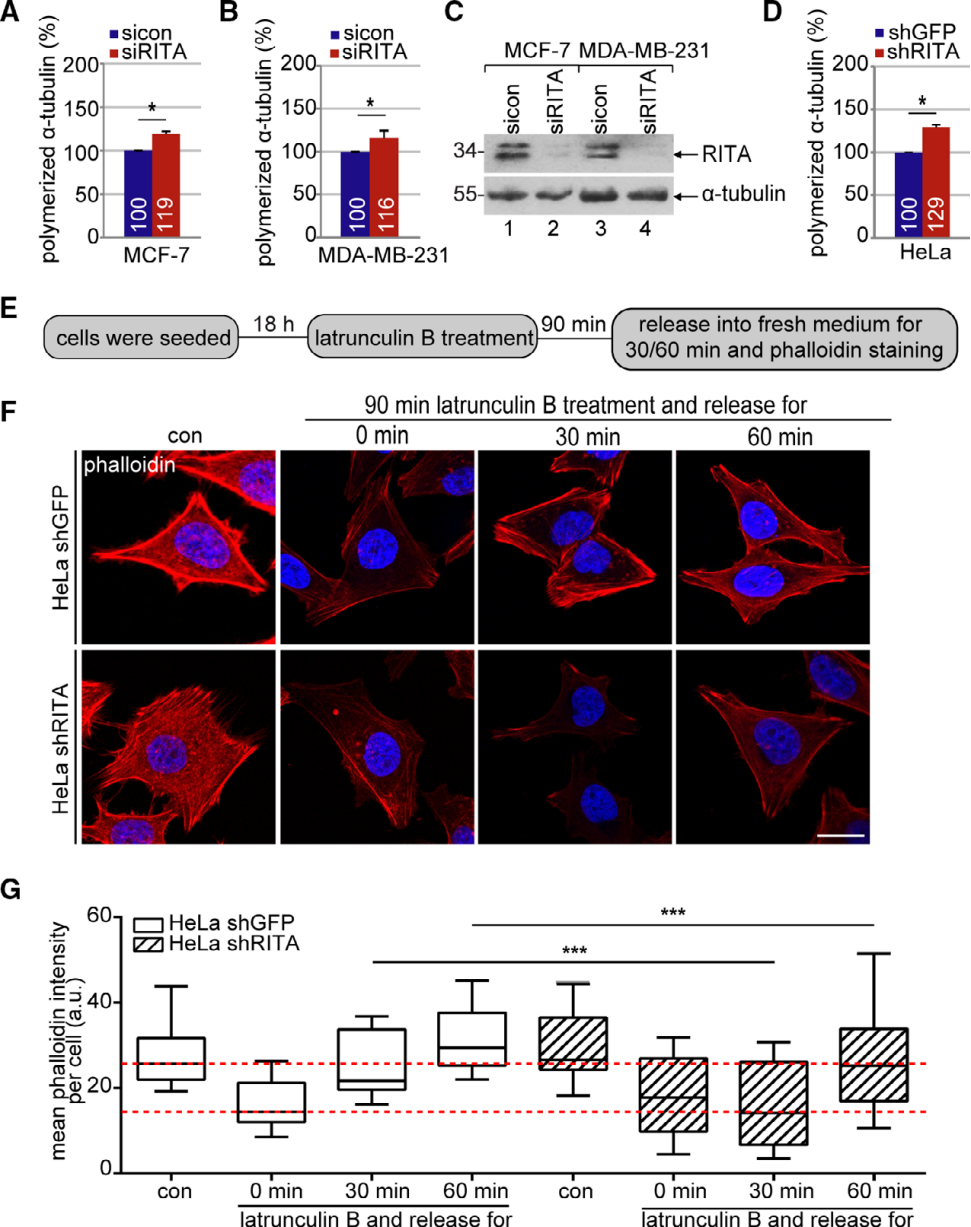
Increased polymerized α‐tubulin and reduced actin repolymerization in RITA‐depleted cancer cell lines. (A, B) MCF‐7 (A) and MDA‐MB‐231 (B) cells were transfected with sicon or siRITA for 48 h. For the measurement of polymerized α‐tubulin *in vivo*, cellular soluble tubulin was pre‐extracted in a MT‐stabilizing buffer. Resuspended cells were fixed and stained for α‐tubulin with a specific mouse monoclonal antibody and FITC‐conjugated rabbit anti‐mouse antibody. The α‐tubulin content of cells was analyzed using a FACSCalibur^TM^. The content in control siRNA transfected cells was assigned as 100%. The results are based on three independent experiments and presented as mean ± SEM. *P < 0.05. (C) Western blot analysis showing the efficient knockdown of endogenous RITA in MCF‐7 and MDA‐MB‐231. α‐tubulin served as loading control. (D) The α‐tubulin content was also measured in HeLa shGFP and shRITA cells. The results are based on three independent experiments and presented as mean ± SEM. *P < 0.05. (E) Schedule of latrunculin B washout experiment. HeLa shGFP and shRITA cells were treated with 10 µm latrunculin B for 90 min and released into fresh medium for indicated time periods. (F) The cells were stained for F‐actin (phalloidin, red) and DNA (DAPI, blue). Representatives of actin fiber reassembly are shown. Scale: 25 µm. (G) Quantification of the mean fluorescence intensity of F‐actin (phalloidin) per cell (10 cells per condition). The results are based on three independent experiments and presented as box plots with bars indicating variations. ***P < 0.001. a.u., arbitrary units. Student’s *t*‐test for (A, B) and (D). Unpaired Mann–Whitney *U*‐test for (G).

Recently, we showed that depletion of RITA leads to stabilized and less dynamic MTs during mitosis (Steinhauser *et al.*, 2017). To explore whether RITA influences MT dynamics during the interphase, MCF‐7 and MDA‐MB‐231 cells depleted of RITA (Fig. 5C) were stained for α‐tubulin for quantitative evaluation via flow cytometry. In both cell lines, knockdown of RITA caused an elevated amount of polymerized α‐tubulin compared to control cells (Fig. 5A,B). This was further corroborated with HeLa shGFP and HeLa shRITA cells (Fig. 5D). These findings support a role for RITA in the orchestration of MT stability during the interphase, which might influence FA dynamics, as MTs often appear associated with FAs regulating their dynamics (Stehbens and Wittmann, 2012).

The actin cytoskeleton, working in concert with FAs, is essential for cell migration (Gardel *et al.*, 2010). To explore whether RITA impacts the actin cytoskeleton, HeLa shGFP and shRITA cells were treated with latrunculin B, a reversible actin monomer‐sequestering agent that blocks fast actin polymerization and abolishes stress fiber formation (Itoh *et al.*, 2005). After 90 min, cells were released into fresh medium for indicated time points and stained for F‐actin with phalloidin (Fig. 5E). The dynamics of the actin polymerization upon latrunculin B release in HeLa shRITA cells differed from that in HeLa shGFP cells (Fig. 5F). Microscopic evaluation showed that HeLa shGFP cells were capable of depolymerizing F‐actin upon latrunculin B treatment with efficient repolymerization at 30 min and regaining the previous F‐actin content at 60 min after the release (Fig. 5G). In contrast, HeLa shRITA cells were unable to re‐polymerize actin fibers at 30 min and showed less F‐actin even at 60 min after the release, though these cells responded well to the treatment of latrunculin B and depolymerized their F‐actin fibers (Fig. 5G). Collectively, these results strongly suggest that the reassembly dynamics of the actin cytoskeleton are compromised in cells depleted of RITA.

### 3.5 Delayed cell spreading with reduced FA formation upon RITA knockdown

**Figure 6:**
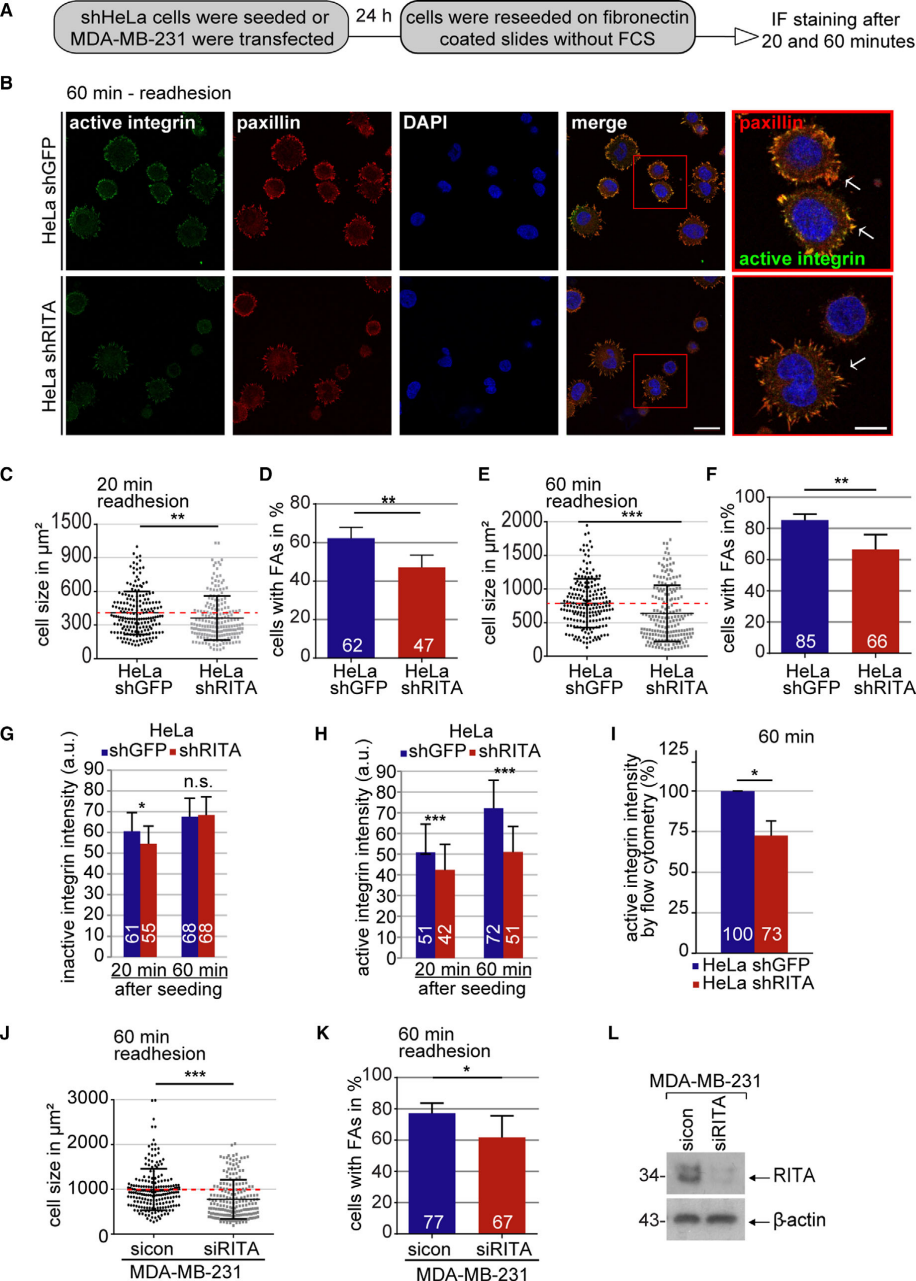
Cell adhesion and integrin activation are compromised upon RITA depletion. (A) Schedule of cell spreading/adhesion assay. (B) HeLa cells stably expressing shGFP or shRITA were stained for active integrin (green), paxillin (red), and DNA (DAPI, blue). Representatives of 60‐min cell re‐adhesion are shown. Scale: 25 µm; inset scale: 10 µm. (C) Quantification of cell size of HeLa shGFP and shRITA cells 20 min after reseeding. The results are based on three independent experiments and presented as scatter plots with bars indicating varied sizes (136 cells). **P < 0.01. (D) Evaluation of cells with FAs 20 min after reseeding (300 cells). The results are based on three independent experiments and presented as mean ± SEM. **P < 0.01. (E) Quantification of cell size 60 min after reseeding. The results are from three independent experiments and illustrated as scatter plots with bars indicating varied sizes (194 cells). ***P < 0.001. (F) Cells with FAs were counted, and the results from three independent experiments are presented as mean ± SEM (219 cells). **P < 0.01. (G) Quantification of the mean fluorescence intensity of inactive integrin 20 and 60 min after cell reseeding (images not shown; 20 min, 195 FAs; 60 min, 205 FAs). The results are based on three independent experiments and presented as mean ± SEM. *P < 0.05. a.u., arbitrary units. (H) Quantification of the mean fluorescence intensity of active integrin 20 and 60 min after cell reseeding. The results are based on three independent experiments and presented as mean ± SEM. ***P < 0.001. a.u., arbitrary units. (I) Active β1‐integrin was also measured via flow cytometry (30 000 cells) 60 min after cell reseeding. The results are based on three independent experiments and presented as mean ± SEM. *P < 0.05. (J) Quantification of MDA‐MB‐231 cell size 60 min after reseeding. The results from three independent experiments are illustrated as scatter plots with bars indicating varied sizes (120 cells). ***P < 0.001. (K) MDA‐MB‐231 cells with FAs were counted, and the results from three independent experiments are presented as mean ± SEM (150 cells). *P < 0.05. (L) Control western blot analysis showing the efficient knockdown of endogenous RITA in MDA‐MB‐231 cells. β‐actin served as loading control. Unpaired Mann–Whitney *U*‐test for (C), (E), and (J). Student’s *t*‐test for (D), (F–I), and (K).

To further clarify whether RITA interferes with FA formation, well‐established cell spreading assays were performed (Lilja *et al.*, 2017). HeLa shGFP and HeLa shRITA cells were trypsinized and reseeded in medium without FBS on fibronectin‐coated slides for 20 and 60 min (Fig. 6A). Cells were stained for active integrin and paxillin (Fig. 6B). The signals of these two FA proteins in HeLa shGFP cells appeared much stronger than in HeLa shRITA cells (Fig. 6B). Cell size, a marker for cell spreading, and the percentage of cells containing FAs, a capability indicator for cell re‐adhesion, were evaluated. The analysis showed that, compared to HeLa shGFP cells, both cell size and number of cells containing FAs were significantly reduced at 20 min (Fig. 6C,D) and 60 min as well (Fig. 6E,F) in HeLa shRITA cells. They displayed nearly the same percentage of cells with FAs at 60 min after reseeding as shGFP cells at 20 min (Fig. 6D,F). This was accompanied by reduced active integrin in HeLa shRITA relative to HeLa shGFP cells at both time points (Fig. 6H), whereas inactive integrin levels remained relatively comparable between both cell lines (Fig. 6G). Reduced active integrin was also detected by flow cytometry (Fig. 6I). Similar results were obtained with MDA‐MB‐231 cells depleted of RITA (Fig. 6J–L), whereas the mRNA levels of integrin were hardly altered upon depletion of RITA (Fig. S3H,I). In summary, RITA knockdown limits integrin activation, compromises FA formation, and inhibits cell spreading.

### 3.6 RITA precipitates with LPP

**Figure 7:**
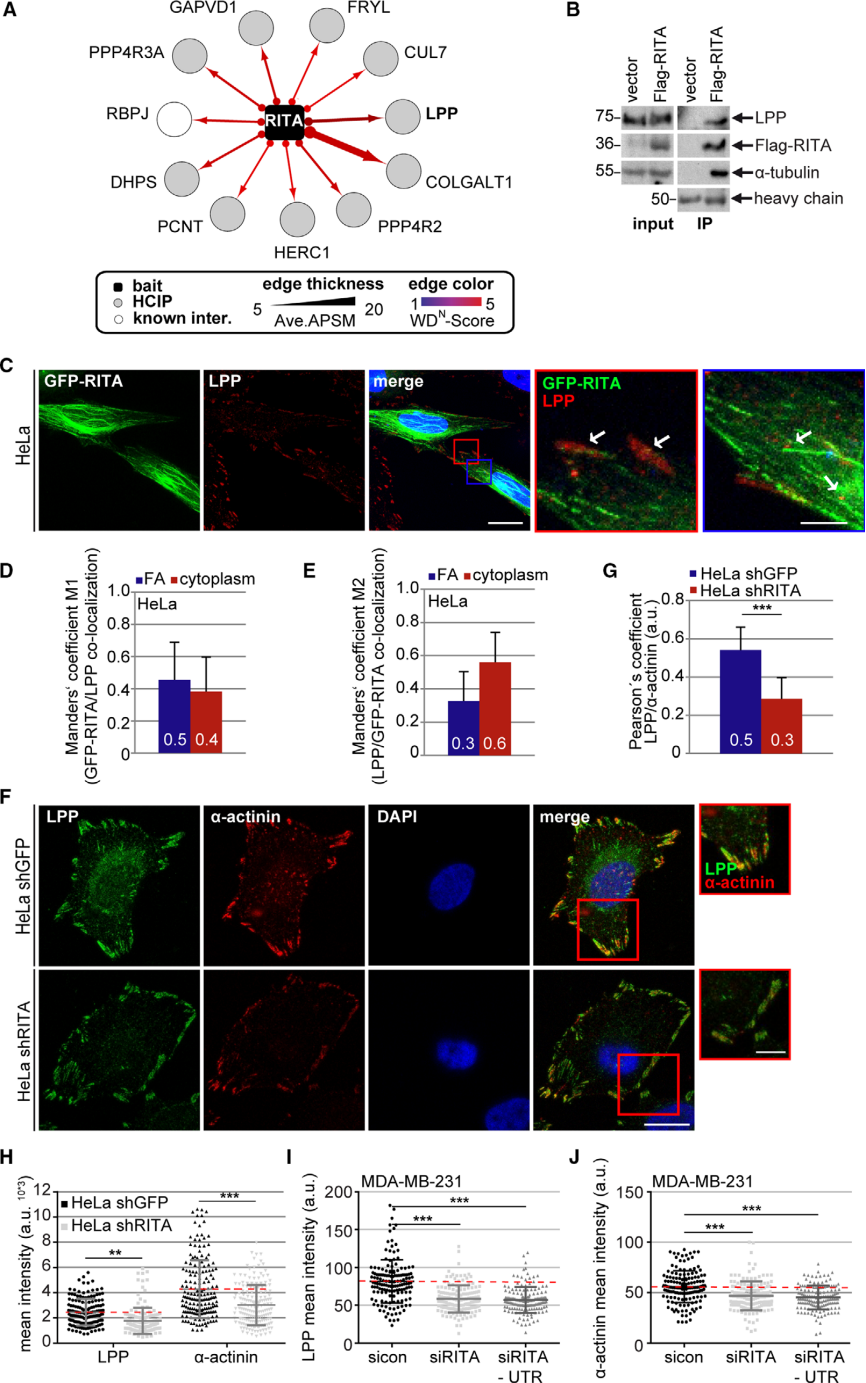
RITA interacts with LPP and its depletion reduces FA‐associated LPP and α‐actinin levels. (A) High‐confidence RITA interaction partners revealed by IP‐MS. HCIP, high‐confidence interaction partners (Ave. APSM ≥ 5 and WD^N^ score ≥ 3). Ave. APSM, average peptide spectral matches. (B) HeLa cells transfected with Flag empty vector or Flag‐RITA were harvested for IP with Flag affinity gel (beads). The precipitates were analyzed by western blot with indicated antibodies. Left panel: input control. Tubulin served as loading control. Right panel: Western blot analysis for IPs. Heavy chain served as precipitate loading control. (C) HeLa cells were transfected with GFP‐RITA (green) and stained for LPP (red) and DNA (DAPI, blue). Representative images are shown. Scale: 20 µm. Inset scale: 5 µm. (D, E) Quantification of the co‐localization with Manders’ coefficients M1 (refers to the green channel; D) and M2 (refers to the red channel; E) represents the correlation between the intracellular locations of GFP‐RITA and LPP (ROI size = 0.25 µm^2^; 50 ROIs). (F) HeLa shGFP or shRITA cells were stained with indicated antibodies for fluorescence microscopy. Representative images are shown. Scale: 12.5 µm; inset scale: 5 µm. (G) Quantification of the co‐localization with Pearson’s coefficient is based on three independent experiments and presented as mean ± SEM. ***P < 0.001. (H) Quantification of the mean fluorescence intensity of LPP (green) and α‐actinin (red). The results are from three independent experiments (LPP, 250 FAs; α‐actinin, 225 FAs) and presented as scatter plots with bars indicating varied intensities. **P < 0.01, ***P < 0.001. a.u., arbitrary units. (I, J) MDA‐MB‐231 cells were treated with sicon, siRITA, or siRITA‐UTR for 48 h and stained for LPP, α‐actinin, and DNA for microscopic evaluation. Quantification of the mean fluorescence intensity is shown for LPP (I) and α‐actinin (J). The results are from three independent experiments (LPP, 250 FAs; α‐actinin, 225 FAs) and presented as scatter plots with bars indicating varied intensity ranges. ***P < 0.001. a.u., arbitrary units. Student’s *t*‐test for (G). Unpaired Mann–Whitney *U*‐test for (H–J).

To investigate whether RITA interacts with proteins of the adhesome, MS analysis was performed with HEK293 cells transfected with hemagglutinin (HA)‐tagged RITA. Lysates were subjected to anti‐HA IP, and HA peptide elution was followed by LC‐MS/MS on trypsinized immune complexes. Total spectral counts for each of the unique proteins were processed using the computational CompPASS as described (Behrends *et al.*, 2010). This analysis revealed several HCIPs (Ave. APSM ≥ 5 and WD^N^ score ≥ 3; Fig. 7A). One of them is RBP‐J, a known interaction partner of RITA (Wacker *et al.*, 2011). In addition, the previously identified tubulins (Steinhauser *et al.*, 2017; Wacker *et al.*, 2011), like tubulin α1C and tubulin β3, were also present though below the strict score threshold (Ave. APSM: 19, WD^N^ score: 1.31, for each tubulin).

We focused on one of the high score partners, the LPP, as it is highly implicated in actin cytoskeleton remodeling, cell migration, and metastasis (Ngan *et al.*, 2018). Moreover, α‐actinin 1, the partner of LPP crucial for FA maturation and cell migration (Foley and Young, 2014), was among the RITA interaction partners (Ave. APSM: 9, WD^N^ score: 2.25). To corroborate the interaction of RITA with LPP, cellular lysates from HeLa cells transfected with control vector or Flag‐RITA were prepared for IP. HeLa cells were efficiently transfected with Flag‐RITA (Fig. 7B, left panel, input control, 2nd row). In the precipitate from RITA‐expressing cells, Flag‐RITA co‐precipitated efficiently with LPP as well as with α‐tubulin (Fig. 7B, right panel, 1st and 3rd row). To look at their subcellular localization, HeLa cells were transfected with GFP‐RITA and stained for LPP (Fig. 7C). GFP‐RITA coated MTs as reported (Steinhauser *et al.*, 2017). The intracellular localization of GFP‐RITA and LPP was further analyzed with the Manders’ coefficients M1 (green to red) and M2 (red to green) (Manders *et al.*, 1993), which is used when the examined proteins strongly differ in their intensities (Zinchuk *et al.*, 2007). M1 and M2 coefficients indicate an actual overlap of two fluorescence signals and represent their degree of co‐localization. A coefficient of 0.5 implies that 50% of both selected channels co‐localize (Zinchuk *et al.*, 2007). GFP‐RITA and LPP co‐localized at FAs (Fig. 7C, red inset magnifications and white arrows) as well as in the cytoplasm (Fig. 7C, blue inset magnifications and white arrows) of HeLa cells. At FAs, there was a co‐localization of 30–50%, whereas in the cytoplasm, we observed 40–60% co‐localization of these two proteins (Fig 7D,E).

### 3.7 RITA depletion results in reduced levels of LPP and α‐actinin at FAs

To determine whether RITA depletion affects the subcellular localization of LPP and α‐actinin, HeLa shGFP and HeLa shRITA cells were stained for these two proteins (Fig. 7F). Their co‐localization at FAs was analyzed with the Pearson’s correlation coefficient, where 1.0 equates to total positive linear correlation (Fig. 7G). The co‐localization coefficient of these two proteins was 0.5 in HeLa shGFP cells, whereas it decreased to 0.3 in HeLa shRITA cells. Additional analysis showed that the mean intensities of both LPP and α‐actinin were significantly reduced at FAs of HeLa shRITA cells (Fig. 7H). These results were further underscored with MDA‐MB‐231 cells transiently depleted of RITA using siRITA or siRITA‐UTR (Figs 7I,J and S3H).

**Figure 8:**
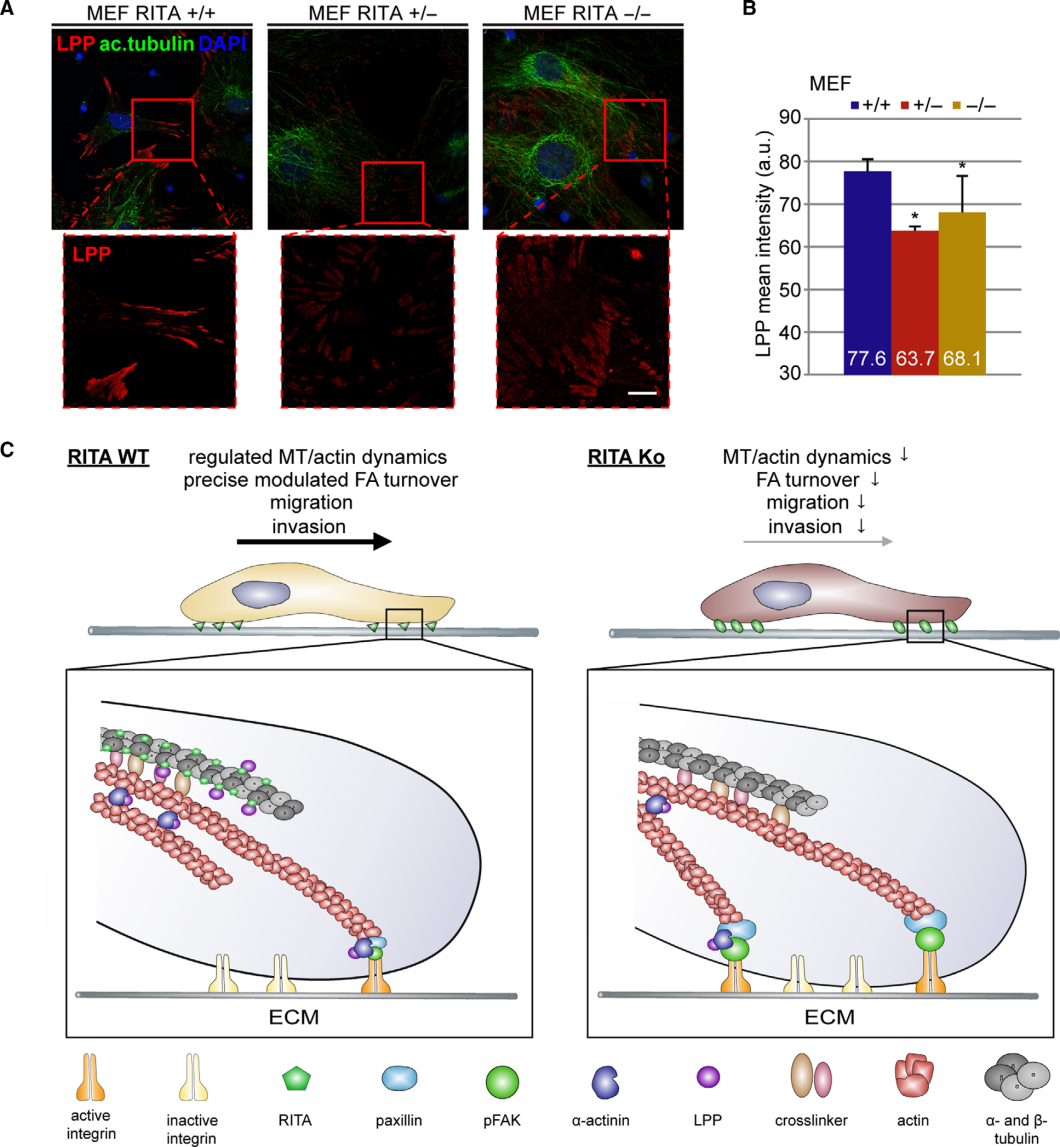
LPP is diminished in RITA knockout MEFs. (A) Wild‐type (RITA^+/+^), heterozygous (RITA^+/−^), and homozygous (RITA^−/−^) MEFs were stained for LPP (red), acetylated tubulin (ac.tubulin, green), and DNA (DAPI, blue) for fluorescence microscopy. Representatives are shown. Scale: 25 µm; Inset scale: 10 µm. (B) The mean fluorescence intensity of LPP of three independent experiments (190 FAs) was quantified and presented as mean ± SEM. Student’s *t*‐test. *P < 0.05. (C) Schematic illustration of the working mechanisms. The presence of RITA ensures regulated MT/actin dynamics and FA turnover (left panel). The absence of RITA impairs MT dynamics and FA assembly/disassembly. Moreover, depletion of RITA reduces the amounts of FA‐associated LPP and α‐actinin interfering with the regulation of the actin cytoskeleton. This leads to compromised cell migration and invasion (right panel).

To further corroborate these results, RITA^+/+^, RITA^+/−^, and RITA^−/−^ MEFs were stained for LPP and acetylated tubulin. Interestingly, relative to RITA wild‐type MEFs, RITA^+/−^ and RITA^−/−^ MEFs displayed a decrease in the LPP signal (Fig. 8A). Further evaluation revealed that the intensity of LPP decreased by 18% in RITA^+/−^ and 12% in RITA^−/−^ MEFs (Fig. 8B). These results strongly indicate that RITA is involved in the regulation of the actin cytoskeleton and FA dynamics by modulating the interaction with LPP.

## 4 Discussion

In the present study, we have identified RITA as a novel modulator of cell motility. Reduced expression of RITA decreases cell migration and invasion attributed to impaired FA turnover linked to compromised actin and MT dynamics.

pFAK, active integrin, and paxillin, the three important FA proteins, were increased in cells depleted of RITA. Though FAK/pFAK is generally considered as a positive regulator of migration, emerging data also demonstrate its negative regulatory role (Zheng and Lu, 2009). FAK together with paxillin inhibited cell migration and their knockdown led to increased motility in HeLa cells and fibroblasts (Schaller, 2004; Yano *et al.*, 2004). Whereas the phosphorylated FAK at Y397 was necessary for the FA formation and signaling, its dephosphorylation was prerequisite for FA disassembly (Zheng and Lu, 2009) and cell migration (Zhang *et al.*, 2006). These data suggest that the timely regulation of FAK is of vital importance for proper FA turnover and efficient cell migration. This supports our finding that increased pFAK, active integrin, and paxillin, induced by depletion of RITA in diverse cancer cell lines and MEFs, disrupt FA dynamics and cause its delayed turnover resulting in reduced migration. These data highlight an involvement of RITA in the regulation of cell motility by modulating FA dynamics. Moreover, FA size is related to cell migration (Kim and Wirtz, 2013) and the residence time of FAK and paxillin increases with FA size (Le Devedec *et al.*, 2012). Compared to control cells, depletion of RITA leads to enlarged FAs in all tested cancer cell lines and MEFs, attributable to disrupted FA disassembly. This enhances the amounts of pFAK and paxillin at FAs, and reduces cell migration and invasion.

Suppression of RITA significantly reduces MT dynamics in mitotic cells (Steinhauser *et al.*, 2017). We show here that RITA depletion increases polymerized MTs in interphase cells. It is well established that MT dynamics is crucial for FA disassembly (Efimov *et al.*, 2008; Kaverina *et al.*, 1999). Thus, RITA could affect FA disassembly by affecting MT dynamics. Additionally, the data from MS suggest that RITA interacts with Cul7, an important regulator of MT dynamics (Yan *et al.*, 2014), which could further strengthen the role of RITA in MT dynamics. Moreover, MT‐mediated vesicle trafficking regulates FA dynamics by affecting critical events like integrin endocytosis (Stehbens and Wittmann, 2012), which could be responsible for observed increased cell surface integrin. Cell motility is also depending on the precise coordination between the MT and actin cytoskeleton (Stehbens and Wittmann, 2012). RITA depletion compromises this coordination demonstrated by delayed actin stress fiber formation. These observations highlight the requirement of dynamic MTs, which are impaired by RITA suppression, for an effective FA disassembly and cell migration.

Most importantly, we show that RITA precipitates with LPP and presumably also with α‐actinin. LPP and α‐actinin play critical roles in actin cytoskeleton regulation and cell migration (Ngan *et al.*, 2018). Loss of LPP decreased cell migration (Vervenne *et al.*, 2009). Moreover, LPP was identified as an interaction partner of the PR130 subunit of catalytically active phosphatase 2A (PP2A) at FAs (Janssens *et al.*, 2016). Decreased LPP induced by depletion of RITA may reduce the recruitment of PP2A, which could lead to sustained pFAK within FAs. While LPP in complex with α‐actinin enhanced cell migration by promoting FA turnover, loss of LPP or deletion of the α‐actinin binding region reduced this capability (Ngan *et al.*, 2013). In addition, α‐actinin regulates FA disassembly through interaction with the protease calpain promoting cell migration (Franco and Huttenlocher, 2005; Raynaud *et al.*, 2003). Interestingly, both LPP/α‐actinin and their co‐localization are reduced at FAs in RITA‐depleted tumor cells and MEFs. This could further contribute to impaired FA disassembly and compromised dynamics of the actin cytoskeleton, leading to reduced migration and invasion of RITA‐deficient cells.

## 5 Conclusion

This study identifies RITA as an important modulator of cell migration and invasion. RITA exerts this function possibly by affecting several critical cellular events including FA dynamics through finely regulated FA proteins, the actin cytoskeleton elasticity via the association with LPP/α‐actinin, and the regulation of MT stability (Fig. 8C, left panel). RITA’s depletion impairs these functions leading to reduced FA turnover, compromised cell spreading, migration, and invasion (Fig. 8C, right panel). Further investigations are required to explore the precise molecular mechanisms underlying our observations.

## Conflict interest

The authors declare no conflict of interest.

## Author contributions

SCH investigated, wrote and reviewed, and edited; AR conceptualized, investigated, wrote and reviewed, and edited; KS and SR investigated; FO and CB investigated and resourced; CS and FL analyzed the data; NNK wrote original draft, investigated, supervised, and conceptualized; JY conceptualized, supervised, performed the funding acquisition, critically wrote and reviewed, and edited.

## Supporting information


**Fig. S1**. MDA‐MB‐231 and MCF‐7 cells lacking RITA exhibit defects in cell migration and overexpression of RITA does not change the migration behavior of MCF‐7 cells.

**Fig. S2**. Motility is reduced in MDA‐MB‐231 cells depleted of RITA and cell viability is hardly changed upon RITA depletion or overexpression.

**Fig. S3**. Functional characterization of HeLa cells stably expressing shGFP or shRITA.

**Fig. S4**. Depletion of RITA enhances the amount of pFAK in MCF‐7 cells and attenuates the MT‐induced FA disassembly in MDA‐MB‐231 cells.
