# Supplementary figures and images for: RITA modulates cell migration and invasion by affecting focal adhesion dynamics
Source: Mol Oncol. 2019 Aug 6;13(10):2121–41. doi: 10.1002/1878-0261.12551 (PMC6763788; doi:10.1002/1878-0261.12551)

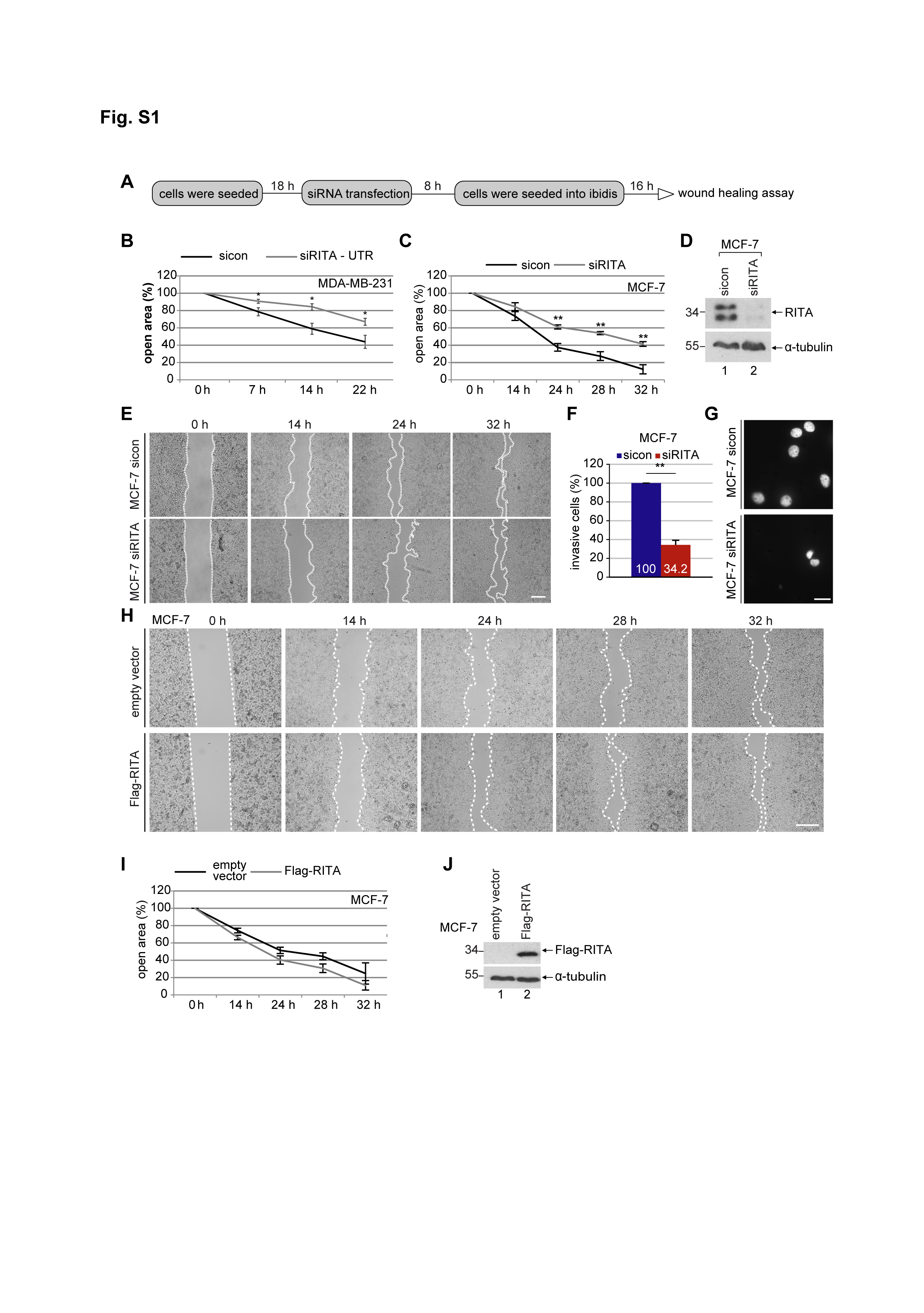

Supplement: Supplementary file 1 — Fig. S1. MDA‐MB‐231 and MCF‐7 cells lacking RITA exhibit defects in cell migration and overexpression of RITA does not change the migration behavior of MCF‐7 cells. [file MOL2-13-2121-s001.tif]

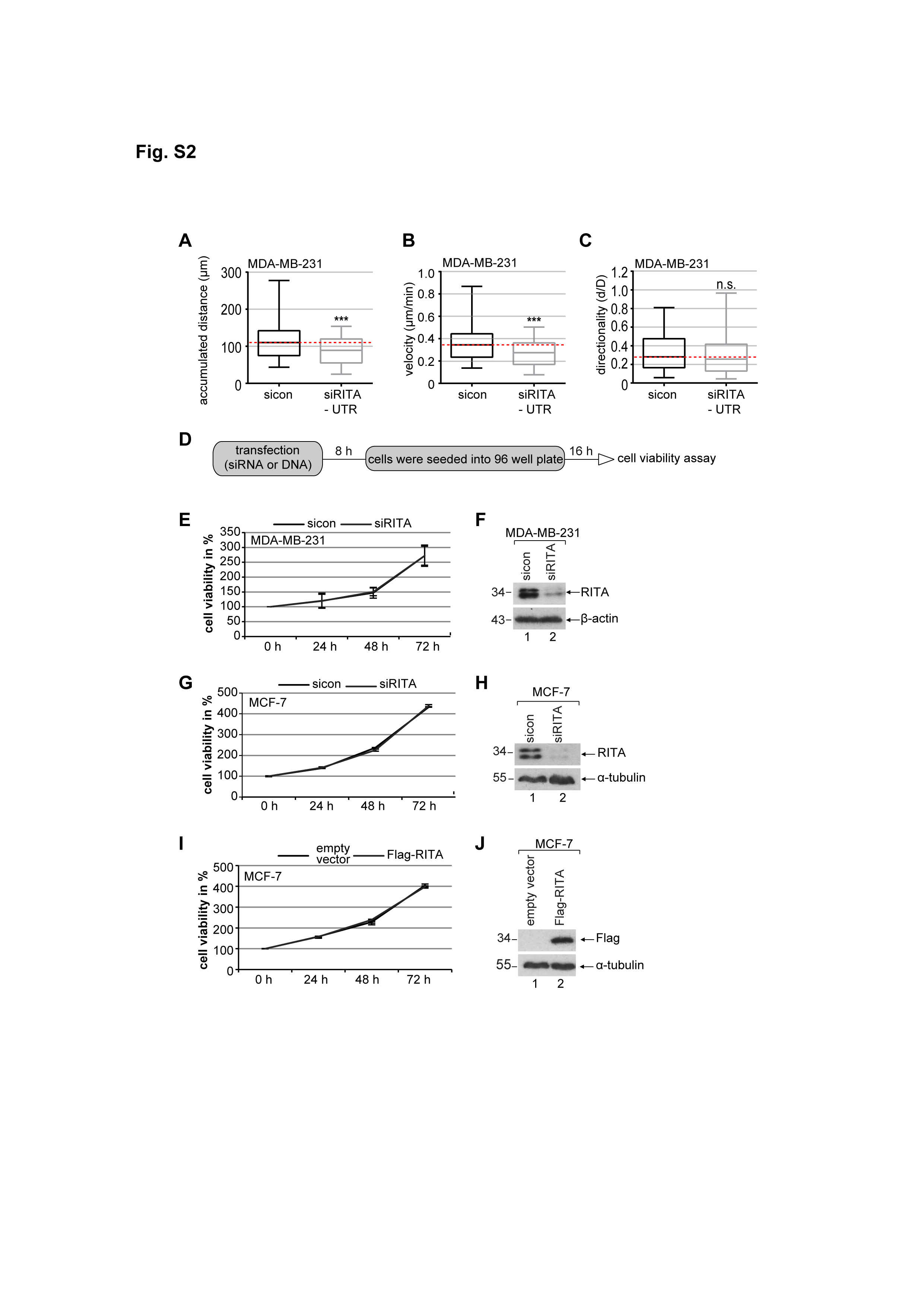

Supplement: Supplementary file 2 — Fig. S2. Motility is reduced in MDA‐MB‐231 cells depleted of RITA and cell viability is hardly changed upon RITA depletion or overexpression. [file MOL2-13-2121-s002.tif]

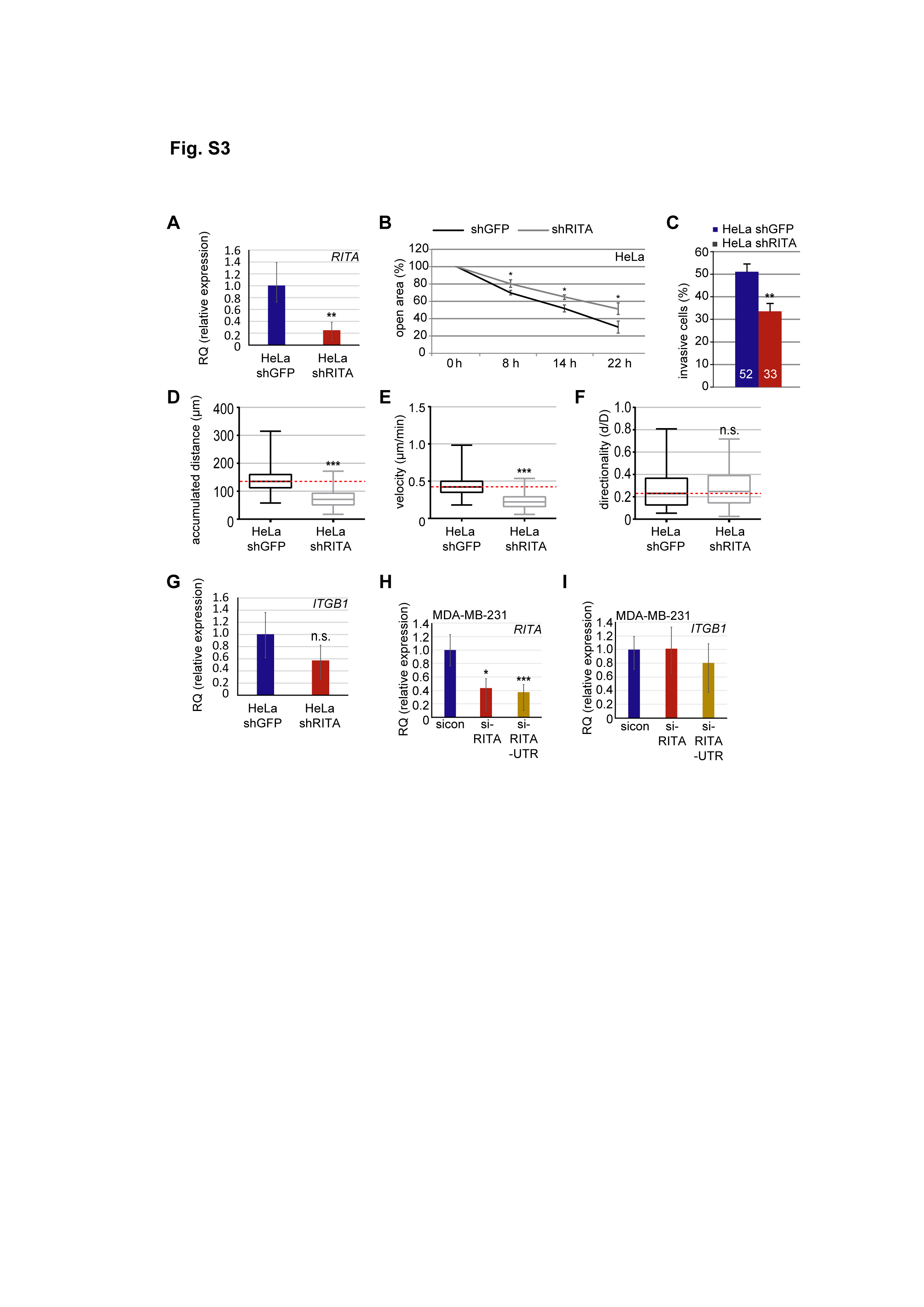

Supplement: Supplementary file 3 — Fig. S3. Functional characterization of HeLa cells stably expressing shGFP or shRITA. [file MOL2-13-2121-s003.tif]

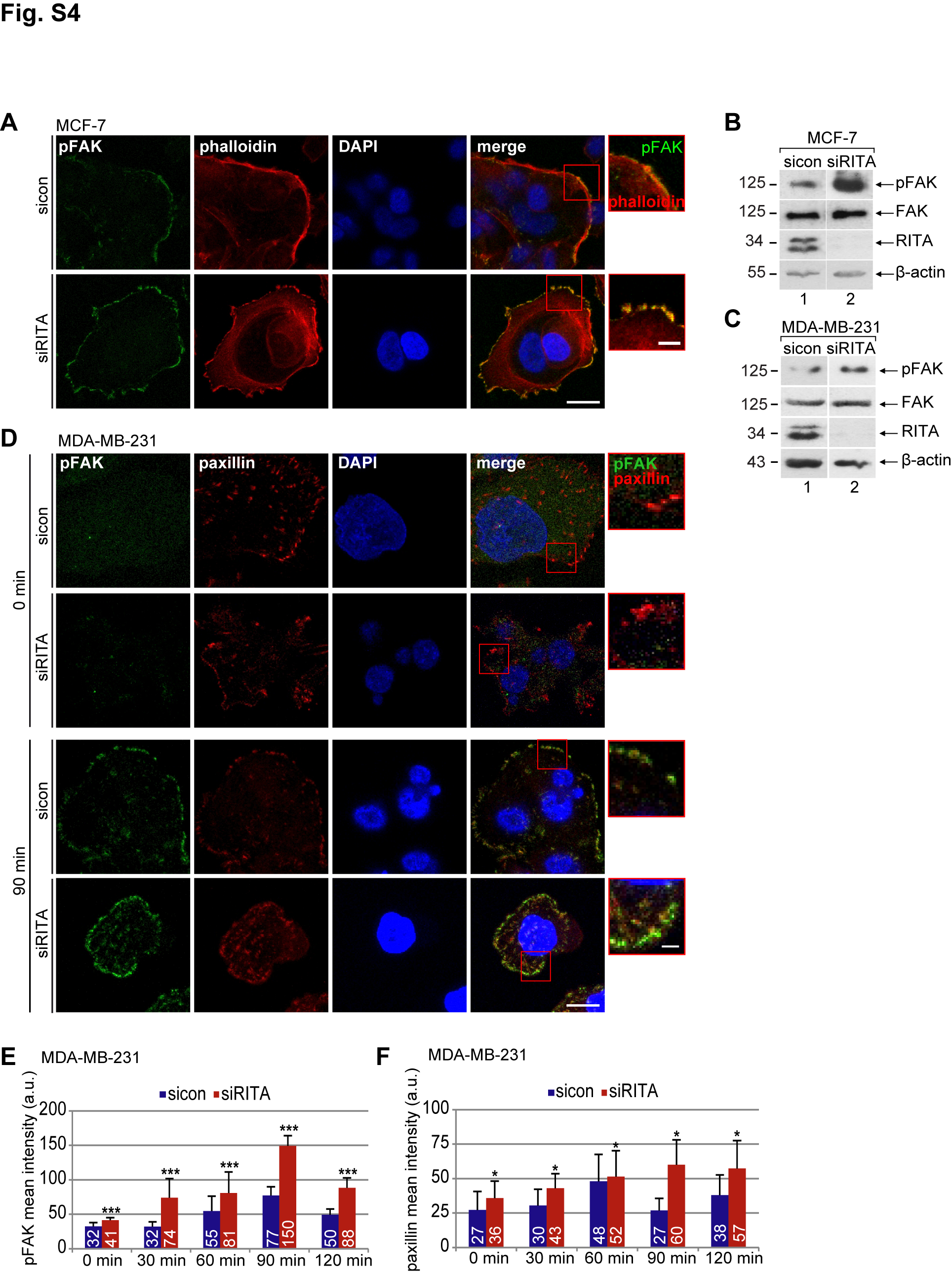

Supplement: Supplementary file 4 — Fig. S4. Depletion of RITA enhances the amount of pFAK in MCF‐7 cells and attenuates the MT‐induced FA disassembly in MDA‐MB‐231 cells. [file MOL2-13-2121-s004.tif]
