# Supplementary material for: RITA modulates cell migration and invasion by affecting focal adhesion dynamics
Source: Mol Oncol. 2019 Aug 6;13(10):2121–41. doi: 10.1002/1878-0261.12551 (PMC6763788; doi:10.1002/1878-0261.12551)
Supplement: Supplementary file 5 [file MOL2-13-2121-s005.docx]

**Supplementary information**

**Fig. S1.** MDA-MB-231 and MCF-7 cells lacking RITA exhibit defects in cell migration and overexpression of RITA does not change the migration behavior of MCF-7 cells. **(A)** Schedule of wound healing/migration assay. **(B and C)** Wound healing/migration assays were performed with MDA-MB-231 (B) cells treated with control siRNA (sicon) or siRNA targeting the 3' untranslated region of RITA (siRITA-UTR) and with MCF-7 cells (C) treated with sicon or siRNA targeting the coding region of RITA (siRITA) and images were taken at indicated time points to document the migration front. The open areas were evaluated between both migration fronts at various time points. The cell-free area at 0 h was assigned as 100%. The results from three independent experiments are presented as mean ± SEM. *p < 0.05, **p < 0.01. **(D)** Western blot transfection control of MCF-7 cells. α-tubulin served as a loading control. **(E)** Images were taken from MCF-7 cells treated with sicon or siRITA at indicated time points to document the migration front. Representatives are shown. White dashed lines depict the migration fronts. Scale: 200 μm. **(F and G)** Invasion assay. MCF-7 cells were treated with sicon and siRITA, seeded into transwell systems and starved for 12 h. The cells were released into fresh medium for 24 h and afterwards fixed. Representatives of invaded MCF-7 cells are shown (G). Scale: 25 μm. The total number of invasive cells in the control group was assigned as 100%. The results from three individual experiments are presented (F), as mean ± SEM. **p < 0.01. **(H)** Wound healing assays were performed with MCF-7 expressing control empty vector or Flag-RITA. Images were taken at indicated time points to document the migration front. Representatives are shown. White dashed lines depict the migration fronts. Scale: 300 μm. **(I)** Quantification of the open area between both migration fronts at various time points. The cell-free area of each individual condition at 0 h was assigned as 100%. The results are based on three independent experiments. **(J)** Cellular lysates were prepared for Western blot analyses with indicated antibodies as transfection efficiency control. α-tubulin served as loading control. Student’s t-test for (B), (C), (F) and (I).

**Fig. S2.** Motility is reduced in MDA-MB-231 cells depleted of RITA and cell viability is hardly changed upon RITA depletion or overexpression. **(A-C)** Time-lapse microscopy was performed with sicon or siRITA-UTR treated MDA-MB-231 cells for up to 12 h. Random motility of these cells was analyzed. Representative trajectories of individual cells (n=30) are analyzed. Evaluated accumulated distance (A), velocity (B) and directionality **(C)** from three independent experiments are shown as box plots with variations. Unpaired Mann-Whitney *U* test. ***p < 0.001. **(D)** Schedule of cell viability assay. **(E)** Cell viability of MDA-MB-231 transfected with sicon or siRITA was measured via CellTiter-Blue^®^ assay. The results from three independent experiments are presented as mean ± SEM and statistically analyzed compared to sicon treated cells with Student’s t-test (not significant, ns). **(F)** Cellular lysates were prepared for Western blot analyses with an antibody against RITA as transfection efficiency control. β-actin served as loading control. **(G)** Cell viability of MCF-7 transfected with sicon or siRITA was measured via CellTiter-Blue^®^ assay. The results from three independent experiments are presented as mean ± SEM and statistically analyzed compared to sicon treated cells with Student’s t-test (ns). **(H)** Cellular lysates were prepared for Western blot analyses with indicated antibodies as transfection efficiency control. α-tubulin served as loading control. **(I)** Cell viability of MCF-7 transfected with control vector and Flag-RITA was measured via CellTiter-Blue^®^ assay. The results are based on three independent experiments and presented as mean ± SEM, statistically analyzed compared to control vector transfected cells with Student’s t-test (ns). **(J)** Cellular lysates were prepared for Western blot analyses as transfection efficiency control. α-tubulin served as loading control.

**Fig. S3.** Functional characterization of HeLa cells stably expressing shGFP or shRITA. (A) Total RNA was extracted from HeLa shGFP and HeLa shRITA cells and the gene levels of *RITA* were measured. The results are based on three experiments and presented as mean ± SEM. **p < 0.01. **(B)** Wound healing/migration assays were performed with these cells and images were taken at indicated time points to document the migration front. The open areas were evaluated between both migration fronts at various time points. The cell-free area at 0 h was assigned as 100%. The results from three independent experiments are presented as mean ± SEM. *p < 0.05. **(C)** Invasion assay. HeLa shGFP or HeLa shRITA cells were seeded into transwell systems and starved for 12 h. The cells were released into fresh medium for 24 h and fixed for quantification. Invaded cells were counted. The total number of invasive cells in the control group was assigned as 100%. The results from three independent experiments are presented as mean ± SEM. **p < 0.01. **(D-F)** Time-lapse microscopy was performed with HeLa shGFP and HeLa shRITA cells for up to 12 h. Random motility of these cells was analyzed. Individual cells (n=30) were analyzed. Evaluated accumulated distance **(D)**, velocity **(E)** and directionality **(F)** from three independent experiments are shown as box plots with variations. ***p < 0.001. **(G)** Total RNA was extracted from HeLa shGFP and HeLa shRITA cells and the gene levels of *ITGB1* were measured. The results are based on three experiments and presented as mean ± SEM. No significant difference could be observed between HeLa shGFP and HeLa shRITA cells. **(H)** Total RNA was isolated from treated MDA-MB-231 cells and the gene levels of *RITA* were measured. The results are based on three experiments and presented as mean ± SEM. *p < 0.05, ***p < 0.001. **(I)** Total RNA was isolated from treated MDA-MB-231 cells and the gene levels of *ITGB1* were measured. The results are based on three experiments and presented as mean ± SEM. No significant difference could be observed between cells treated with sicon and with siRITA/siRITA-UTR. Student’s t-test for (A), (C) and (G-I). Unpaired Mann-Whitney *U* test for (D-F).

**Fig. S4.** Depletion of RITA enhances the amount of pFAK in MCF-7 cells and attenuates the MT-induced FA disassembly in MDA-MB-231 cells. **(A)** MCF-7 cells were treated with sicon and siRITA and stained for pFAK (green), phalloidin (red) and DNA (blue) for fluorescence microscopy. Representatives are shown. Scale: 12.5 µm; inset scale: 5 µm. **(B)** Western blot analysis of MCF-7 cells. β-actin served as loading control. **(C)** Western blot analysis of MDA-MB-231 cells. β-actin served as loading control. **(D)** MDA-MB-231 cells were transfected with sicon and siRITA. The cells were incubated for 5 h with 10 µM nocodazole followed by washout, whereby the MTs were allowed to regrow for the indicated time points. Cells were stained for pFAK (green), paxillin (red) and DNA (blue). Representatives of FA reassembly are shown. Scale: 12.5 µm, inset scale: 5 µm. **(E-F)** Quantification of the mean fluorescence intensity of pFAK **(E)** and paxillin **(F)** (70 FA per condition in a defined ROI). The results are based on three independent experiments and presented as mean ± SEM. Student’s t-test for (E and F). *p < 0.05, ***p < 0.001.
